# Roles of researchers in inter- and transdisciplinary sustainability research: a reflection tool

**DOI:** 10.1007/s11625-024-01619-x

**Published:** 2025-02-17

**Authors:** Benjamin Hofmann, Hanna Salomon, Sabine Hoffmann

**Affiliations:** 1 Department of Environmental Social Sciences, Eawag: Swiss Federal Institute of Aquatic Science and Technology, Überlandstrasse 133, 8600 Dübendorf, Switzerland; 2 Department of Environmental Systems Science, IED, ETH Zurich, Universitätsstrasse 16, 8092 Zurich, Switzerland; 3 Department of Environmental Systems Science, TdLab, ETH Zurich, Universitätsstrasse 16, 8092 Zurich, Switzerland

**Keywords:** Roles of researchers, Tool, Inter- and transdisciplinary research, Sustainability science, Operationalization, Reflection

## Abstract

Inter- and transdisciplinary (ITD) research is increasingly called for and supported to promote sustainable transformation through knowledge co-production, knowledge integration, and solution development. The paper explores what is needed to support researchers in reflecting on their new roles in ITD research. We introduce a reflection tool that makes the growing literature on researchers’ roles in sustainability science applicable to ITD projects. Its design is based on the arguments that each researcher can have several roles within one research project and that focusing on a few key roles increases clarity compared to differentiating many specialized roles. The tool consists of (1) a researcher survey that operationalizes six prominent roles (traditional scientist, self-reflexive scientist, knowledge integrator, knowledge broker, process facilitator, and change agent), (2) a visualization of role profiles from the survey, and (3) a set of reflection questions on related opportunities, challenges, and coping strategies on individual and project level. We empirically applied the tool in two ITD research projects focused on sustainable food production and water and forest ecosystem management. Comparative application of the tool yielded diverse role profiles of researchers in both projects, with different patterns for senior and junior researchers as well as natural and social scientists. The reflection produced a collection of opportunities, challenges, and coping strategies that corroborates and extends insights from ITD literature. We discuss how the tool triggers multi-dimensional reflection on roles (individual and project level, role combinations, self-perception and external perception) and outline opportunities for further strengthening such reflection in ITD research.

**Supplementary Information:**

The online version contains supplementary material available at 10.1007/s11625-024-01619-x.

## Introduction

The increasingly recognized need for sustainable transformation in many parts of society has given rise to new forms of science and knowledge production (Schoolman et al. 2012; Lang et al. 2012; Fazey et al. 2018; Norström et al. 2020; Caniglia et al. 2021). One of these forms is inter- and transdisciplinary (ITD) research that has been called for increasingly, among others, by “[p]olicymakers on the European and national levels, funding agencies, and many academic institutions” (Felt 2022, p. 204; cf. Glänzel and Debackere 2022). ITD research enables researchers from different disciplines as well as stakeholders from policy and practice to grasp the complexity of sustainability problems by crossing the boundary between their different values, concepts, and perspectives (Nurius and Kemp 2019; Pohl et al. 2021; Hoffmann et al. 2022). Core aspects of ITD research are knowledge co-production, knowledge integration, and solution orientation (Lang et al. 2012; Miller et al. 2014; Hoffmann et al. 2017a). Knowledge integration refers to “a process of combining a wide range of perspectives from different disciplines (i.e., interdisciplinary integration), as well as from research, policy, and practice (i.e., transdisciplinary integration)” (Hoffmann et al. 2022, p. 2) and to its outputs (O’Rourke et al. 2016). Knowledge co-production denotes “iterative and collaborative processes involving diverse types of expertise, knowledge and actors to produce context-specific knowledge and pathways towards a sustainable future” (Norström et al. 2020, p. 183). Solution-oriented research in the field of sustainability “investigates actions and practices… that are intended to advance sustainable development” (Lang and Wiek 2022, p. 31).

A precondition for successful knowledge integration, knowledge co-production, and solution development is that researchers (and stakeholders) move beyond their traditional roles and take on new and/or different roles within the ITD team. Such new roles are usually shaped by contextual conditions and either adopted purposefully (‘role-taking’) or gradually in an interplay of the integration, co-production, and solution context and the personal qualities and competencies of researchers and stakeholders (‘role-making’) (Hilger et al. 2021; Hoffmann et al. 2024). They are not static and may change over time (Huning et al. 2021; Arnold 2022). Some of the roles, such as ‘traditional scientist’ align well with the roles researchers generally adopt, while others like ‘knowledge broker’ or ‘change agent’ transcend existing roles (Hoffmann et al. 2024). Being aware of the existence of these different roles in ITD teams helps to make their own and others’ expectations about the different roles transparent (Bulten et al. 2021; Hilger et al. 2021). This, in turn, can help to address or prevent potential challenges or conflicts arising from certain roles or role combinations (Hilger et al. 2021). Further, it can scale down expectations that individual researchers and stakeholders need to perform all roles to co-produce and integrate new knowledge and develop solutions (Hilger et al. 2021). Therefore, it is important that researchers of ITD teams reflect on their roles and the challenges and opportunities they entail.

In this paper, we explore what is needed to support researchers in making use of the growing literature on the roles they can and do play in ITD research projects. We argue that, for making this body of knowledge relevant for the practice of sustainability science, researchers from various fields need to be enabled to understand and reflect on roles in a simple, accessible, and time-efficient manner. More specifically, we see five objectives for enhanced reflection: (1) foster researchers’ individual awareness of their own roles; (2) explore the relationship between researchers’ different roles in terms of opportunities, challenges, and coping strategies; (3) enable an exchange between their own and external perceptions of researchers’ roles within a team; (4) map the combination of researchers’ roles on the project level; and (5) spark discussion about how well this combination of roles aligns with the project goals. These five objectives have guided us in the development of a reflection tool that could be integrated into existing toolkits for ITD research (cf. Laursen et al. 2024). The reflection tool is anchored in the literature on researchers’ roles, notably in debates about whether researchers can have only one or different roles within an ITD project (e.g., Pielke 2007; Crouzat et al. 2018; Bulten et al. 2021) and about the number and granularity of roles that should be differentiated (e.g., Wittmayer and Schäpke 2014; Horlings et al. 2020; Bulten et al. 2021; Hilger et al. 2021). Our aim was to design a tool that reaches the five objectives for enhanced reflection while being sufficiently parsimonious for application across a wide range of ITD research projects.

The paper is structured as follows: first, we review major theoretical perspectives on roles of researchers and justify the design choices of our reflection tool. Second, we describe the tool’s method and its empirical application in two ITD research projects. Third, we present the data obtained from this application, highlighting what preliminary role patterns, opportunities, challenges, and coping strategies emerged. Fourth, we discuss our results against the backdrop of ITD literature and with a focus on the tool’s contribution to the broader knowledge base. Fifth, we conclude with ideas on how to further advance reflection on researchers’ roles in ITD research.

## Theory

### Perspectives on the roles of researchers

The roles of researchers have received increasing attention in the scholarly literature in recent years. Many scholars have outlined different ideal–typical roles that researchers can or should take on when conducting ITD research and described specific activities related to these roles (e.g., Pohl et al. 2010; Wittmayer and Schäpke 2014; Adelle et al. 2020; Bulten et al. 2021; Hilger et al. 2021; Kruijf et al. 2022; Hoffmann et al. 2022; Schrage et al. 2023). As research on transformations combines questions of ‘what is’ and ‘what ought to be’ and devotes particular attention to ‘what can be’ (Avelino and Grin 2017), the spectrum of potential roles in this field is broad, encompassing analytical, pragmatic, as well as normative orientations. While tools exist to reflect on research stances (Hazard et al. 2020) and to explore identities of researchers (Temper et al. 2019), we argue that there is still a lack of an easily applicable tool that allows for mapping the roles of researchers in ITD research and reflecting on opportunities and challenges that these role profiles entail on individual and project level. Such reflection is important as it can support a reflexive approach to the position of sustainability science (e.g., inquiries into values and desirable futures) (Miller et al. 2014; Fazey et al. 2018), shed light on links between researchers’ roles and goals of ITD research (e.g., solution-oriented and process-first projects) (Miller 2013; Miller et al. 2014; Lang and Wiek 2022), and problematize external constraints for certain roles (e.g., in terms of institutional structures and career prospects) (Miller et al. 2011; Guimarães et al. 2019).

The literature provides different entry points for reflection on researchers’ roles. One debate is whether researchers need to decide for a single role or can play multiple roles within the duration and context of one research project. Pielke (2007) assumes that researchers need to choose from the four mutually exclusive roles of pure scientist, science arbiter, honest broker of policy alternatives, and issue advocate, even though roles may change throughout a career. Scholars have mapped this distinction on a role continuum (Donner 2014) and differentiated the range of possible roles further, but maintained the idea that researchers need to choose one role (Crouzat et al. 2018, pp. 99–103). The value of this conceptualization lies in its parsimony and its ability to trigger debate about the relationship between science and advocacy. By contrast, another strand of literature argues that roles can be combined (Huning et al. 2021), even though tensions may arise between certain roles (Wittmayer and Schäpke 2014; Arnold 2022). Bulten et al. (2021) study the relationship between role combinations on the level of individual researchers. They find that while most roles are compatible or mutually supportive, certain combinations are problematic. For example, they find that the role of a transition leader clashes with the roles of traditional and self-reflexive scientists; and the process facilitator role conflicts with the traditional researcher role, too (Bulten et al. 2021, p. 1278). Research has shown that even rather different boundary-spanning roles, such as science arbiter, honest broker of policy alternatives, and issue advocate, can be combined by the same researchers in the same process (Sarkki et al. 2020). Likewise, the repertoires of some allegedly “new” knowledge brokering roles are surprisingly aligned with more “traditional” roles of scientists (Turnhout et al. 2013).

Another relevant debate is about how many different role profiles are needed to adequately capture the different activities of researchers. There is one part of the literature that focuses on further differentiating researchers’ roles into ever more fine-grained profiles. One example is Hilger et al. (2021) who identified 15 roles that researchers and practitioners take on. Through this detailed analysis, the authors show “the breadth of activities” (Hilger et al. 2021, p. 2064) that characterizes transdisciplinary sustainability research. Scholars have developed even more fine-grained typologies for specific roles, such as different types of transition intermediaries (Kivimaa et al. 2019), roles of researchers in collaborative governance interventions (Peltola et al. 2023), different profiles in knowledge transfer (Thompson et al. 2006), and various types of transformative and activist researchers (Temper et al. 2019). Another part of the literature describes a more limited number of researchers’ roles (Pohl et al. 2010; Wittmayer and Schäpke 2014; Horlings et al. 2020; Bulten et al. 2021). Several well-known role labels, such as self-reflexive scientist or change agent, have emerged from this literature and serve as focal points for identity formation among sustainability scientists.

### Towards a reflection tool

The two debates inform the basic design of a tool for fostering reflection about researchers’ roles in ITD projects. We hold that an approach that allows for role combinations is most conducive to reflection. While some researchers may identify primarily with one specific role, typologies with mutually exclusive roles are too simplistic as they disregard the possibility of playing multiple roles within one research project. First, they paint with a broad brush over the diversity of roles found in scientific practice, ignoring, for instance, different degrees of reflexivity in scientists’ work (Popa et al. 2015). Second, their preference for honest brokerage neglects that there is a wider spectrum of roles available for scientists who engage with society, including, for example, the facilitation of processes that empower stakeholders to participate in knowledge co-production and co-learning (Roux et al. 2017). Third, their notion of mutually exclusive roles clashes with the reality of many sustainability researchers, which is characterized by their desire—and societal expectations—to contribute to both scientific progress and societal problem-solving (Wittmayer et al. 2017; Fazey et al. 2018). The approach we adopt pays greater attention to the diversity of roles within science and in its engagement with societal stakeholders, is open for incorporating new roles that complement existing roles (e.g., knowledge integration on top of traditional science), and takes into account that sustainability researchers often need to combine roles to create academic and societal impact. Indeed, researchers need to take on a diversity of tasks in different project phases (Stauffacher et al. 2008), resulting in distinct personal journeys within ITD projects (McGowan et al. 2014). Thus, a tool that helps to reflect on various roles that researchers play will have to go beyond a simple continuum.

We further argue that a limited number of roles is best suited for reflection. Fine-grained role differentiation may be useful for understanding specific processes, such as knowledge transfer and uptake in governance interventions and transitions, but is likely too complex for fostering reflection for three reasons. First, a small number of easily understandable and applicable role descriptions supports reflection even among researchers who are unfamiliar with the different ideal–typical roles described in the literature. Second, some of the numerous roles described in the literature overlap in their activities (e.g., ‘knowledge collector’ and ‘scientific analyst’ in Hilger et al. 2021) and/or are located in similar places at interfaces of science and policy or practice (e.g., “intermediary”, “knowledge broker”, and “expert in learning” in Kruijf et al. 2022, p. 398). Third, we argue that facilitation of “expectation management, mutual transparency, and clarity of roles” (Hilger et al. 2021, p. 2066) is better achieved through conceptual parsimony rather than complexity. Bringing the debates about the number and compatibility of roles together, we decided that our reflection tool should do two things: it should allow for a certain complexity by capturing multiple roles that researchers can play over the course of one research project, but limit complexity regarding the number of potential roles covered.

Our tool covers six different roles that researchers can assume within an ITD project team based on existing typologies and descriptions in the literature (Pohl et al. 2010; Wittmayer and Schäpke 2014; Hilger et al. 2018, 2021; Adelle et al. 2020; Bulten et al. 2021; Hoffmann et al. 2022): traditional scientist, self-reflexive scientist, knowledge integrator, knowledge broker, process facilitator, and change agent (see Table 1). Drawing on this literature, we define the roles as follows (cf. Salomon 2023).^1^ The traditional scientist is a scientist who deconstructs complex problems and systematically analyzes and communicates them in an intersubjective way. A self-reflexive scientist observes and critically reflects on research practices, power dynamics, and his/her and the team’s normative orientation. A knowledge integrator uses integrative methods to bridge different perspectives from different disciplines and fields, leading to new integrated knowledge, and assesses and evaluates these processes and the resulting outputs. A knowledge broker identifies and connects relevant actors from science, policy, and practice and adapts and tailors different types of knowledge to specific target audiences. A process facilitator designs the learning process (e.g., by organizing workshops), provides space for critical reflection, and condenses the outcomes. Finally, a change agent strategically networks with actors from science, policy, and practice in the context of change processes by, for example, coaching and advising actors from policy and practice, motivating them to lead change processes, and facilitating and participating in such processes (cf. Miller 2013).

**Table 1:** Operationalization of six roles of researchers in ITD projects

Role		Activities/tasks	Example of conducting activity/task	Adapted from
1	Traditional scientist	Deconstruct complex problems into solvable parts	Apply scientific concepts and theories	Adelle et al. (2020, p. 58) and Bulten et al. (2021, p. 1273)
		Conduct or supervise the conduct of systematic analyses of deconstructed problems and potential solutions	Use quantitative and/or qualitative methods	Bulten et al. (2021, p. 1273), Pohl et al. (2010, p. 277) and Wittmayer and Schäpke (2014, p. 488)
		Communicate scientific knowledge validated as intersubjective/objective by the respective discipline	Write peer-reviewed scientific publications, give presentations at scientific conferences	Bulten et al. (2021, p. 1273) and Pohl et al. (2010, p. 276)
2	Self-reflexive scientist	Observe and reflect on research practices	Write observation protocols or keep research diaries	Bulten et al. (2021, p. 1273) and Hilger et al. (2018, p. 143)
		Critically reflect on internal and external power dynamics that shape the project	Identify hierarchies and differences in resource endowment	Bulten et al. (2021, p. 1273) and Wittmayer and Schäpke (2014, pp. 488–489)
		Critically reflect on own normative orientation in relation to project goals	Reflect on own personal motivations, attitudes, and policy preferences	Bulten et al. (2021, p. 1273) and Wittmayer and Schäpke (2014, pp. 488–489)
3	Knowledge integrator	Cross or bridge boundaries of different disciplines or fields	Link theoretical concepts from different disciplines or fields, co-create integrative frameworks, or develop interdisciplinary methods	Hoffmann et al. (2022, p. 3)
		Synthesize knowledge from different disciplines or fields and generate new integrated knowledge	Recognize critical connections and leverage potential synergies	Hoffmann et al. (2017b, p. 680) and Hoffmann et al. (2022, p. 3)
		Design, plan, monitor, assess, and evaluate integrative processes and their integrated outputs	Develop a shared vision for integration, defining who contributes what, at which stage, for which purpose, and supported by which methods and procedures	Hoffmann et al. (2017b, p. 690) and Hoffmann et al. (2022, p. 3)
4	Knowledge broker	Identify and connect relevant actors from science, policy, practice, and/or the public	Map the actor landscape, identify, and mediate different perspectives	Adelle et al. (2020, p. 58), Bulten et al. (2021, p. 1273) and Wittmayer and Schäpke (2014, p. 488)
		Bridge different types of knowledge	Make scientific knowledge usable for different target audiences and/or integrate knowledge from policy, practice, and/or the public into the scientific process	Bulten et al. (2021, p. 1273), Turnhout et al. (2013, pp. 358; 361–362) and Juhola et al. (2024, p. 6)
		Translate, interpret, adapt, and tailor different types of knowledge to different target audiences	Find feasible problem–solution couplings	Adelle et al. (2020, p. 58) and Bulten et al. (2021, p. 1273)
5	Process facilitator	Initiate and facilitate learning processes or experiments within project team and/or with actors from policy, practice, and/or the public	Develop tools to jointly reflect on researchers’ roles and/or integration in the project	Bulten et al. (2021, p. 1273), Wittmayer and Schäpke (2014, pp. 488–489) and Pohl et al. (2010, p. 277)
		Organize and prepare workshops, select and invite actors (from science, policy, practice, and/or the public), and condense the outcomes	Design workshops to develop action options and analyze implications of these options for sustainability transformations	Bulten et al. (2021, p. 1273), Adelle et al. (2020, p. 58) and Wittmayer and Schäpke (2014, p. 488)
		Provide space for critical reflection and deliberation	Encourage expression of different viewpoints	Hilger et al. (2021, p. 2056) and Pohl et al. (2010, p. 277)
6	Change agent	Strategically network with actors from science, policy, practice, and/or the public in the context of change processes	Engage with actors’ concerns and generate possible solutions for real-world problems	Wittmayer and Schäpke (2014, pp. 488–489)
		Intervene into policy, practice, and/or the public with the aim to contribute to change processes	Engage in policy consultations and in innovation processes in relation to practices, contribute to reconfiguration of actor relations	Bulten et al. (2021, p. 1273) and Hilger et al. (2018, p. 143)
		Empower actors from policy, practice, and/or the public to lead own change processes	Make use of motivation and capacity-building tools	Wittmayer and Schäpke (2014, pp. 488–489), Bulten et al. (2021, p. 1273) and Adelle et al. (2020, p. 58)

Compiled by the authors; sources indicated in table

We agree that having multiple, overlapping, or changing roles within a project can lead researchers to “experience tensions between different roles” (Bulten et al. 2021, p. 1279). Such tensions and potential incompatibilities, however, are a matter for empirical investigation rather than conceptual pre-definition. The same applies to the potentially diverse tasks that researchers adopt. For instance, the role of critical scholars may combine tasks from across the roles of self-reflexive scientists (e.g., critically reflect on power dynamics), knowledge integrators (e.g., bridge boundaries of different disciplines or fields), and process facilitators (e.g., provide space for critical reflection and deliberation). Thus, while the role labels provide some orientation, the individual combination of tasks should serve as basis for reflection.

## Method and materials

### Tool design

The reflection tool consists of a role survey for individual researchers, a spider web graph for immediate role visualization on the individual and project level, and a set of questions for individual and project team reflections. All these elements can be implemented in a workshop setting of around one hour. Drawing on existing role descriptions, the role survey operationalizes each of the six roles that we focus on in the form of three typical tasks (see Table 1). A researcher filling out the survey decides for each task whether she/he performs this task in the context of the specific ITD project (assigning it a score of 1) or not (assigning it a score of 0). From these responses, a simple additive score ranging from 0 (= role not assumed) to 3 (= role fully assumed) is calculated. The rationale behind equal weighting of tasks is that any ranking would imply valorizing certain ways of doing research more than others, which we think is not in line with the pluralist community of sustainability science. Once aggregated, the scores for all six roles are transferred into a spider web graph to obtain the individual role profile of the researcher (see Supplementary Material). The reason for choosing a spider web is that it is neutral with respect to combinations of roles. It allows for wearing different hats at the same time, a possibility that has been stressed by other authors but is not well reflected in their tools using one or two axes to map researchers’ roles (cf. Schrage et al. 2023). It also mirrors our position that (in-)compatibilities of different roles should be a subject of empirical inquiry rather than conceptual decision. The individual role profiles can be laid on top of each other in a project-level spider web graph to see which roles are most strongly present within the team and which ones are less represented. A set of questions for individual and project team reflection helps discussing the results (cf. Temper et al. 2019, p. 5), notably opportunities and challenges of different role combinations, potential coping strategies on the individual level (Bulten et al. 2021, pp. 1280–1281), and the alignment of roles performed by the team with the project goals (Table 2). Our incorporation of the project level goes beyond the focus on individual reflection in the existing literature (cf. Kruijf et al. 2022; Schrage et al. 2023).

**Table 2:** Guiding questions for reflection

Level	Question
Individual	1. What opportunities do you experience with respect to your roles (e.g., in terms of synergies, resources, expectations)?
	2. What challenges do you face with respect to your roles (e.g., in terms of tensions between specific roles, resources, expectations)?
	3. What coping strategies have you developed to seize these opportunities or address these challenges?
Project	4. To what extent does the combination of roles in your team fit the project goals?
	5. What opportunities and challenges does the combination of roles in the project entail, and what coping strategies do you see?

Compiled by the authors

### Project context

We applied the tool in two ITD research projects in the area of sustainability. The project “Transformation in Pesticide Governance” (TRAPEGO) explores the potential for an evidence-based sustainable transformation of agricultural pesticide policy and practice (cf. Hofmann et al. 2023). It employs an ITD approach to understand the preferences and interactions of different policy and practice actors in an area of “societal, economic, and environmental trade-offs” (Ingold et al. 2024). The project “Transformation Toward Resilient Ecosystems: Bridging Natural and Social Sciences” (TREBRIDGE) aims to identify policy and management approaches for alpine ecosystems to increase their resilience taking into account societal needs regarding natural resource use and protection. Similar to TRAPEGO, it pursues an ITD approach, which is seen as critical for project success since “the complex, crosscutting, and multi-faceted nature of ecosystems cannot be tackled adequately by a single discipline or disconnected approaches” (Lieberherr et al. 2021, p. 5).

The two projects are similar in many other respects, including their geographical focus, duration, and funding source (see Table 3). Both ITD projects have a separate work package dealing with science integration and aiming to bridge the various work packages within the projects. Furthermore, both projects aim for knowledge integration and solution development. To do so, they pursue disciplinary publications within separate work packages alongside integrated publications in inter- and transdisciplinary journals as well as in the form of policy briefs and other practice outputs. In addition, both projects organize, facilitate, and participate in a series of workshops to envision pathways to sustainability. This combination bridges the dichotomy between knowledge-first and process-oriented projects (Miller 2013). Almost all researchers in the two projects are affiliated with public universities or research institutes.^2^ Both projects also involve societal stakeholders, for example, through an advisory board. These similarities allow for a meaningful comparison across the two ITD projects.

**Table 3:** Comparison of ITD research projects in which tool was applied

	TRAPEGO Transformation in Pesticide Governance	TREBRIDGE Transformation Toward Resilient Ecosystems
Empirical research focus	Agricultural pesticide use	Alpine watershed management
Geographic focus	Switzerland	Switzerland
Project duration	2021–2025	2022–2026
Budget	CHF 2.8 million	CHF 2.3 million
Funding source	Swiss National Science Foundation: Sinergia program	Swiss National Science Foundation: Sinergia program
Number of research institutes	5	4
Total number of researchers in project*	17	15
Thereof		
Seniors (professors, group leaders)	9	10
Juniors (postdocs, PhDs, scientific assistants)	8	5
Thereof		
Natural scientists (incl. public health researchers)	8	8
Social scientists	9	7
Disciplines or fields involved	Agronomy, agricultural economics, decision analysis, environmental sciences, political science, public health, and inter- and transdisciplinary studies	Environmental economics, forest ecology, geology, geomorphology, hydrology, natural resource policy, and inter- and transdisciplinary studies
Number of stakeholders in advisory board	16	12
Thereof		
Public administration (national)	4	3
Public administration (subnational)	2	6
Private sector	6	0
Research and civil society	4	3
Knowledge co-production	In sub-parts of the project, e.g., collection of best practices of evidence, design of questions in farmer surveys, and assessment of pesticide policy options	Across entire project, including development of scenarios, prioritization thereof, and acceptance of policy and management options for the different scenarios
Knowledge integration	Integrated understanding of role of evidence for sustainable policy and practice, especially impact of evidence on attitudes of actors in Swiss agriculture towards pesticide use and regulation	Holistic understanding of the functioning of geomorphic processes in water and forest ecosystems; insights on plural valuation of nature (including non-monetary values)
Solution development	Policy and practice options for reducing environmental and human health risks of agricultural pesticides	Policy and management options for enabling transformation towards resilient water and forest ecosystems
Societal context of project	Conflictive: actor coalitions with competing policy preferences	Cooperative: ‘coalitions of the willing’ committed to project goals

*At time of role workshop. Compiled by the authors

Differences between the projects can be found in the natural and social science disciplines involved and in the societal context of the issue investigated. Importantly, while both projects are clearly interdisciplinary, they differ in their degree of transdisciplinarity. Each project features an advisory board that, through annual meetings, has provided stakeholder feedback on the planned research activities and their political and/or practical relevance. In TRAPEGO, knowledge co-production with stakeholders has been used only in specific parts of the project. Based on their experience and expertise, stakeholders identified best practices of evidence use in pesticide governance, provided feedback on the design of survey questionnaires, and were crucial informants to assess the impacts of different policy and practice options in a multi-criteria decision analysis. By contrast, in TREBRIDGE, knowledge co-production has been an integral part of the overall project design, as a participatory scenario development process together with stakeholders is at the heart of the project. For example, stakeholders identified together with researchers key parameters for future scenarios (e.g., context factors, drivers of change) and co-developed four plausible scenarios for three case study regions. They will also assess policy and management options under different scenarios toward the end of the project. Overall, the moderate variance in project characteristics provides a basis for empirical insights that can inform hypothesis-building beyond a single project context.

The design and application of the reflection tool has been part of our accompanying research in these two ITD projects. The aim of this accompanying research has been to produce integrated knowledge about researchers’ roles in both ITD projects, which Defila and Di Giulio (2018) describe as ‘meta-type research’. This meta-type research has aimed for exploring and analyzing researchers’ role(s) as well as for feeding the findings back to the project team to enable it to adapt processes, if necessary. In doing so, we have taken on a dual role of traditional scientists and process facilitators (Salomon 2023). As traditional scientists, we have designed and implemented the reflection tool to explore researchers’ roles in both ITD projects. As process facilitators, we have enabled critical reflection on researchers’ roles at individual and project level that have triggered some adaptations in both projects. This dual role has allowed us to conduct research on researchers’ roles with in-depth knowledge of both projects and has provided a rich basis for interpretation of workshop results, including self-reflection and reflection of others on our own roles (cf. Hoffmann et al. 2017a; Verwoerd et al. 2020).

### Data collection and analysis

In both ITD projects, the application of the reflection tool focused on the researchers and made use of the same workshop setting for data gathering (see Supplementary Material). In a first step, we asked the researchers to fill out the role survey individually. The researchers then mapped their individual results on the spider web graph showing which roles each researcher takes up and to what degree. Additionally, we asked the researchers to individually reflect on opportunities and challenges they experience with respect to these roles (e.g., synergies, tensions, resources, expectations) and strategies they might have developed to seize opportunities and/or address challenges. In a second step, we asked the researchers to build breakout groups of five to six people, copy their individual results to a group spider web graph summarizing the combination of roles of that group, and share their results and insights within the groups. As the leaders of science integration in both projects, we moderated this exchange and noted down challenges and opportunities on a flipchart. In a third step, we summarized the breakout group results in the plenum and initiated a discussion about the fit of the combination of roles with the project goals in terms of contributions to academia and societal problem-solving (cf. Salomon 2023). In total, we gathered data from 29 researchers in the two projects (TRAPEGO: 16; TREBRIDGE: 13).^3^ We documented the contents of the group and plenum discussions during the workshops as well as afterwards based on audio recordings and own memories.

We analyzed the data by identifying overarching themes across the two ITD projects and comparing them with each other. Due to differences in internal project timelines, we conducted the workshops at different points in time from the start of each project (TRAPEGO: 19 months; TREBRIDGE: 5 months). As we will discuss later, this imposes some limitations to our comparison but also suggests new hypotheses about changes in researchers’ roles throughout a project. For each project, the theoretical perspectives reviewed above guided the analysis of the role profiles of the researchers, whereas the analysis of their reflection was inductive. After separate analysis for each project, we compared the results with a focus on identifying patterns that could inspire future research.

## Results

This section presents the ex-post analysis of empirical data collected with the role reflection tool from researchers working in the ITD projects TRAPEGO and TREBRIDGE. We compare their role profiles in three dimensions (aggregated role profiles of all researchers, seniority of researchers, and disciplinary affiliation) and summarize the reflection on opportunities, challenges, and coping strategies on individual and project level.

### Comparison of role profiles between projects

**Fig. 1:**
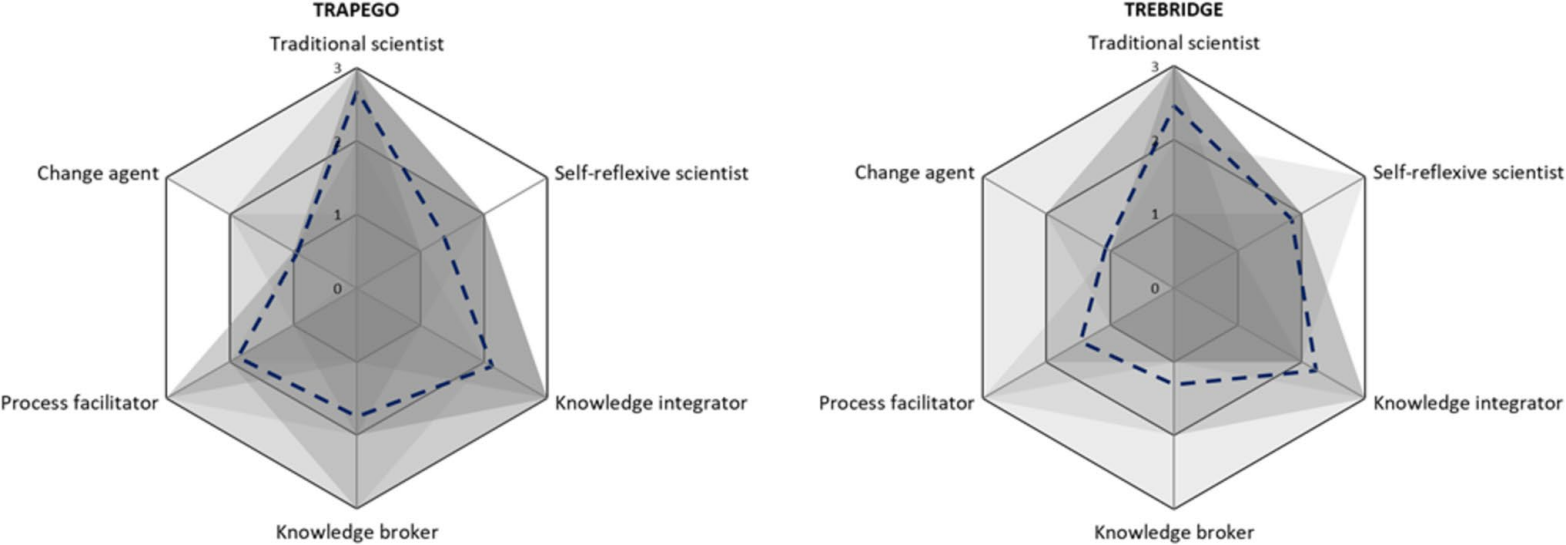
Comparison of role profiles of researchers on project level. The spider webs map the number of tasks taken on by researchers per role. The dotted blue line shows the average number of tasks per role across all researchers. The grey areas show the overlaid role profiles of individual researchers, with darker areas indicating overlap of many researchers and lighter areas indicating less overlap. White areas mean that none of the role profiles covers this role or role combination. Compiled by the authors, for TREBRIDGE: Salomon (2023)

Our first comparison focuses on the aggregated role profiles of all researchers in each project. The role profiles of both projects share several similarities. To begin with, both projects show a broad diversity of roles on project level (see Fig. 1) as well as diverse role combinations on individual level. Among the six roles covered, the traditional scientists is most prominent in both projects. Many scientists are anchored in this role and, on top of it, take on additional roles. This pattern is reflected in the publication strategies of both projects, which mainly target disciplinary outlets complemented with integrated publications in inter- and transdisciplinary journals. The knowledge integrator role is relatively strong in both projects, too, while the change agent role was weakest. Underneath the similar breadth and diversity of roles in both projects, however, we found differences for certain roles. Knowledge broker and process facilitator roles were less strongly represented in TREBRIDGE than in TRAPEGO. Conversely, TREBRIDGE researchers self-identified more strongly with tasks of self-reflexive scientists.

**Fig. 2:**
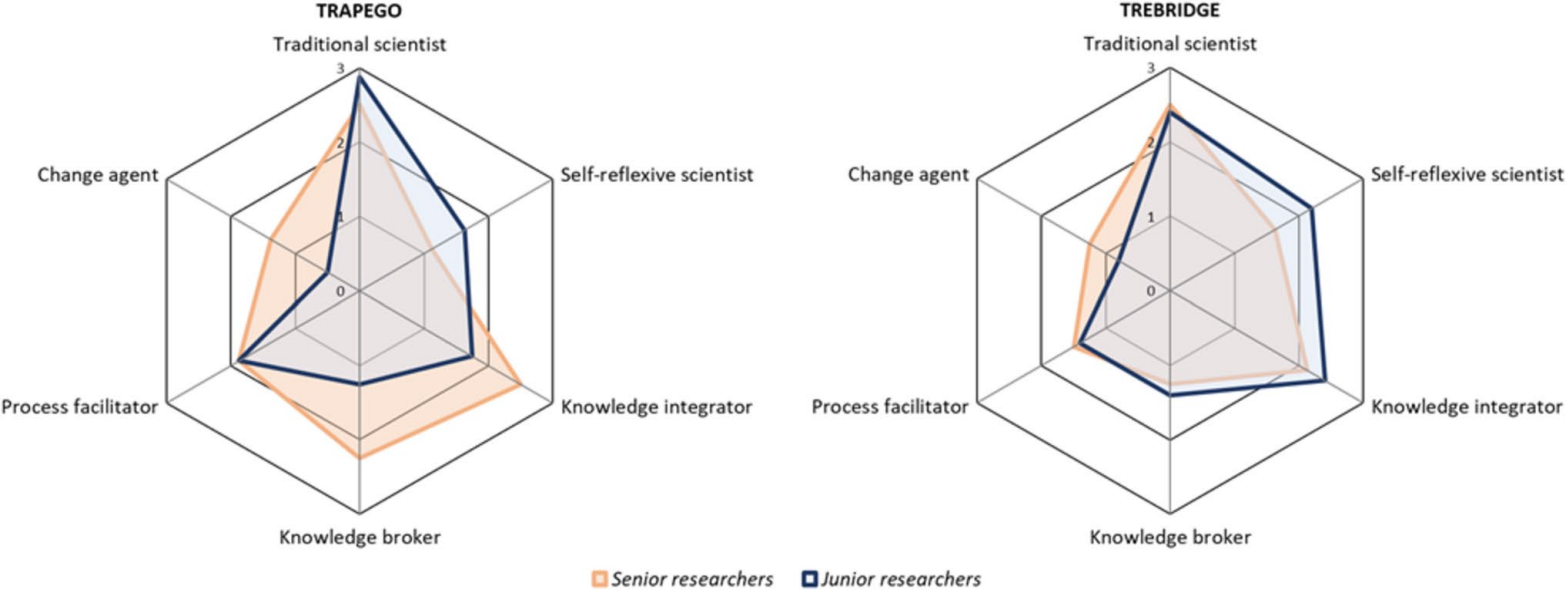
Comparison of role profiles of senior and junior researchers. The spider web maps the average number of tasks taken on by all senior researchers (= orange line) and all junior researchers (= blue line) per role. Compiled by the authors

Our second comparison concerns the role profiles of senior and junior researchers. Senior researchers include professors and group leaders, whereas junior researchers include postdocs, PhDs, and scientific assistants. In both projects, senior researchers performed a higher number of change agent tasks, whereas junior researchers saw themselves more strongly as self-reflexive scientists (see Fig. 2). By contrast, senior and junior researchers had comparable levels of engagement in the roles of traditional scientist and process facilitator. The same applies to knowledge integrator and knowledge broker roles in TREBRIDGE, whereas in TRAPEGO senior researchers engaged more strongly in these roles. The two project leaders, who are part of the senior researcher group, had a very broad role profile. Thus, seniority seems to influence which roles researchers assume, especially in terms of self-reflexivity and pushing for societal change.

**Fig. 3:**
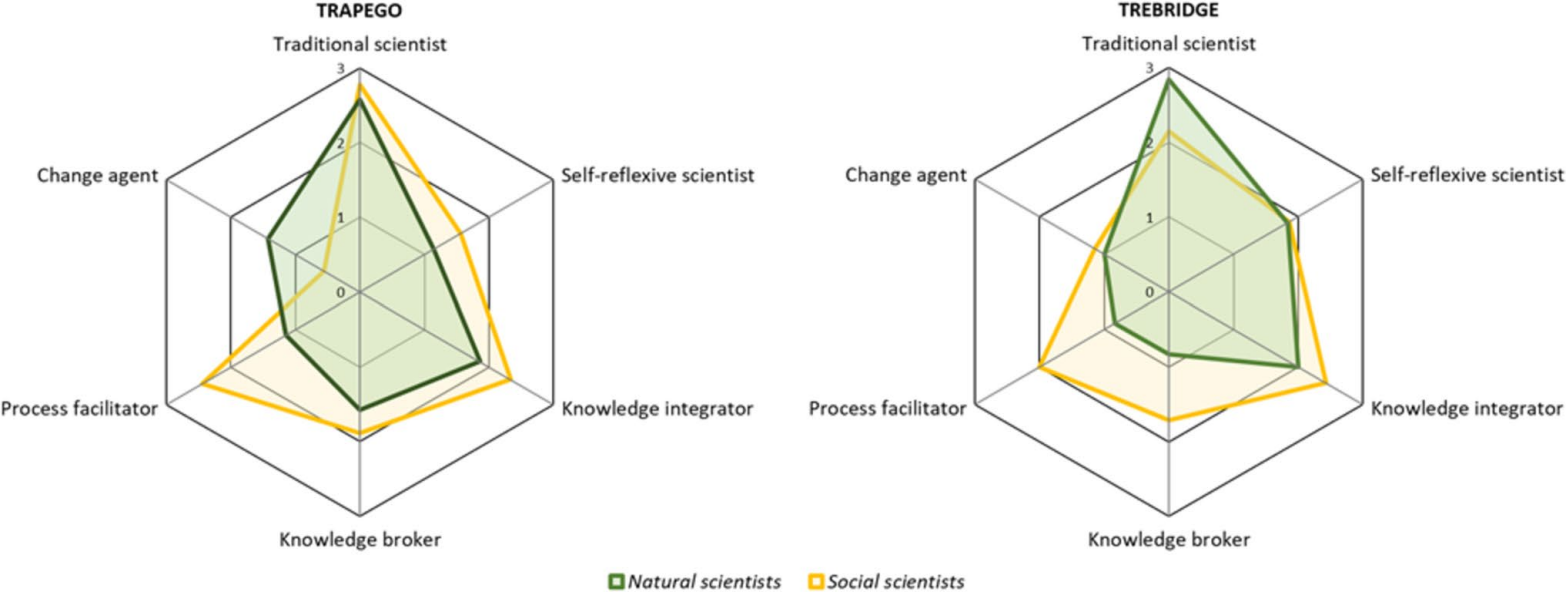
Comparison of role profiles of natural and social scientists. The spider web maps the average number of tasks taken on by all natural scientists (= green line) and all social scientists (= yellow line) per role. For TRAPEGO, natural scientists include public health researchers. Compiled by the authors

Our third comparison is the distinction between natural and social scientists according to researchers’ current department and disciplinary affiliation. In both projects, it was mostly social scientists who performed the three roles of knowledge integrator, knowledge broker, and process facilitator (see Fig. 3). The differences were less clear-cut for the other three roles. Natural scientists (including health scientists) and social scientists took on a comparable level of traditional scientist tasks in TRAPEGO, whereas natural scientists scored higher on this role in TREBRIDGE. For self-reflexive scientist tasks, natural and social scientist were roughly on equal level in both projects. For the change agent role, natural scientists clearly dominated this role in TRAPEGO, whereas in TREBRIDGE natural and social science researchers were on similar levels again. Hence, disciplinary patterns across projects are observed for roles related to integration, brokerage, and facilitation—but not for others.

In summary, researchers assumed a broad diversity of roles in the two ITD projects. Project context as well as distinctions between senior and junior researchers and between natural and social scientists seem to matter for understanding which roles researchers take on.

### Opportunities and challenges of role profiles

The tool allowed for individual and collective reflection on researchers’ own roles and roles of others as well as on the combination of role profiles at project level. A few differences in terms of role perceptions were detected. Some researchers did not think they performed many change agent tasks, whereas their colleagues thought they did, sparking discussion on the extent to which researchers do and should play this role in both ITD projects. Furthermore, the role workshop provided time and space to explore and reflect on the opportunities and challenges that arise when assuming different roles in ITD projects. It also allowed for joint discussion of coping strategies to overcome challenges and leverage opportunities (see Table 4).

**Table 4:** Opportunities, challenges, and coping strategies mentioned during the application of the tool

	Opportunities	Challenges	Coping strategies	Literature related to challenges
*Individual roles*				
Traditional scientist	Deepen one’s specific knowledge in one discipline or field	Get access to field (e.g., for data collection) without strong prior relationships with stakeholders	Set up advisory board with stakeholders to facilitate data access	Robson and McCartan (2016)
Self-reflexive scientist	See the bigger picture and reflect on how and why one does own research	Reserve time to step back and reflect on own research	Build in time at project level for regular reflection from the beginning of the project to the end	Verwoerd et al. (2020)
Knowledge integrator	Learn how to translate own findings into other (inter-)disciplinary languages and connect to other disciplines and fields	Gain recognition for own intellectual contributions	Make own contributions explicit	Ding et al. (2020) and Hoffmann et al. (2022)
Knowledge broker	Open up to different views and ways of thinking (e.g., learning new vocabularies when translating own findings for stakeholders)	Overcome (trans-)disciplinary language barriers when communicating with stakeholders	Learn from fellow researchers and own experience	Nurius and Kemp (2019)
Process facilitator	Enhance competencies that can be employed in different contexts later on	Open up discussions within the whole project team while ensuring autonomy of decision-making within sub-groups	Create moments of self-reflection and joint group reflection to facilitate learning	Bell and Morse (2013)
Change agent	Create possibility for actionable real-life change	Navigate demanding and exhausting power plays	Distribute roles within project team and create institutional support structures (e.g., advisory board with stakeholders)	Wittmayer and Schäpke (2014)
*Combination of roles*				
Knowledge integrator, knowledge broker, and process facilitator	Positive spill-overs between roles, e.g., interactions with researchers and stakeholders in facilitator role support integrator and broker roles	Keep overview of the project’s research and the empirical data gathered by ‘traditional scientists’, and time needed for integration	Attend regular meetings and exchange with ‘traditional scientists’ to keep abreast of project developments	Deutsch et al. (2025a)
Traditional scientist and change agent	Good opportunity to also be a change maker rather than adhering solely to the role of traditional scientist	Run the risk of undermining one’s own legitimacy and reputation as scientist	Make assumptions about role of science in relation to policy and practice explicit as well as ground work in solid science records and methods	Bulten et al. (2021)

Compiled by the authors

In both ITD projects, researchers mentioned two general opportunities related to the project-level role profiles (for TREBRIDGE, cf. Salomon 2023). One opportunity is to have researchers spread across roles within the project team. On individual level, this offers possibilities for exploring different roles within the ITD project and for learning from others, which helps improve own research and its impact. On project level, researchers with different roles can complement each other, supporting division of labor and more focused individual role profiles. Another opportunity is to have a separate work package within the ITD project to ensure reflection on individual and collective role profiles. For instance, making existing role combinations transparent facilitated discussions about spill-overs and (in-)compatibilities between roles. Role diversity is an opportunity provided that researchers are aware of it and actively reflect on it.

A general challenge identified by several researchers was the difficulty of maintaining high engagement in multiple roles in light of time, capacity, and resource constraints. This is most pronounced when own role expectations and project requirements partly diverge. As a coping strategy, participants identified ongoing individual and collective reflection on role profiles (e.g., on whether one has to take on all indicated roles) as well as pragmatism (e.g., consider feasibility and prioritize certain roles). For instance, joint exploration of role profiles in project workshops could be a basis for taking decisions on role distribution within the project team and corresponding resource allocation and capacity-building. In both projects, following application of the reflection tool, researchers with very broad role profiles started to delegate more tasks instead of trying to cover all roles to a high extent themselves. Coping strategies were also devised in relation to specific roles. For instance, self-reflexive scientists indicated the need for more time to step back and reflect on the research process and related power dynamics. Regular meetings and exchange among junior researchers in both projects served as such a reflection space. For us co-authors, who have combined knowledge integrator, knowledge broker, and process facilitator roles, another challenge is that collaborative processes usually take long. The time needed for preparing inter- and transdisciplinary publications clashes with the “publish or perish” imperative that still determines academic career prospects (Purvis et al. 2023). This challenge could best be addressed through changing incentive and reward structures within academia, illustrating the limitations of developing coping strategies on project level (Deutsch et al. 2025b).

## Discussion

The diverse role profiles we mapped reflect the combination of knowledge integration, knowledge co-production, and solution development in both ITD projects, bridging the divide between knowledge-first and process-oriented goals. The results confirm the persistent strength of the traditional scientist role even in ITD research, as research remains anchored in disciplines with ITD research being an add-on (Deutsch et al. 2025b). At the same time, strong presence of the knowledge integrator role in both projects reflects the ITD priority of the funding scheme and the dedicated planning of science integration by senior researchers (Lieberherr et al. 2021; Ingold et al. 2024). According to the discussion in both projects, the weak position of the change agent role may be due to the newness of the role (Hilger et al. 2021), uncertainty on the part of researchers about the role’s legitimacy, perceived reputation risks, and potentially also their affiliation with public research organizations. The reflection about being a change agent suggests that stakes of policy and practice actors influence the roles adopted by them. The conflictive setting of Swiss pesticide policy, with competing coalitions that support or oppose stringent measures, made researchers in TRAPEGO hesitant to embrace this role—a problem not observed in TREBRIDGE, embedded in a more cooperative setting of Swiss water and forest management. Additionally, researchers in both projects emphasized the importance of the time dimension, as role profiles might change. The reflection also raised questions about distributing roles within project teams in a way that ensures division of labor, which deviates from the idea that other roles come on top of the traditional scientist. Such division of labor may reduce tensions experienced by individual researchers, especially project leaders, when taking on many different roles within a project but may create new challenges in ensuring interconnectedness of different roles.

Our results revealed interesting preliminary patterns in role distribution within the ITD project teams. Senior researchers assuming a stronger change agent role may indicate that they possess larger and more sustained stakeholder networks and/or that their more secure academic position allows them to take more time and risks in engaging with policy and practice (Sobey et al. 2013; cf. Evans and Cvitanovic 2018; Guimarães et al. 2019). In turn, stronger self-reflectiveness of junior researchers may show that, as they have not been fully socialized into one discipline or way of doing research yet or are distinctively cross- or undisciplinary (Knaggård et al. 2018; Haider et al. 2018), they still ask more critical questions about why and how to do their own research than senior researchers. Furthermore, the strong reliance on social scientists for taking the roles of knowledge integrator, knowledge broker, and process facilitator may be associated with the skills they bring to the project. All three roles require engagement with social processes that social scientists may have studied as part of their education and training (Ding et al. 2020, p. 7). However, this pattern may change when more dedicated ITD education and training of integration, brokerage, and facilitator skills is offered across different study programs, including the natural sciences (Haider et al. 2018; cf. Horn et al. 2023). The diverse role profiles we mapped underscore the need for a more flexible publication culture and for institutional structures (e.g., incentives, and rewards) that support dynamic and adaptive careers (Miller et al. 2011).

Regarding our goal of enabling researchers to make practical use of the literature on roles in ITD research, the empirical application confirms the value of the proposed reflection tool. First, the tool allowed for detecting a diversity of roles that individual researchers assume, thus providing a richer picture than typologies with mutually exclusive roles. Second, its focus on six roles proved to be a parsimonious design that supported efficient application and balanced role diversity and overlaps. Third, the transparent and multi-dimensional reflection sparked by the tool supports improved collaboration in ITD projects, for instance, by facilitating more conscious decisions about roles. Despite these contributions, we see potential for further developing the tool in different application contexts. To begin with, the set of reflection questions can be expanded depending on the goals of its use in ITD projects (e.g., to enhance role clarity, discuss division of labor, or identify gaps in a project team). Furthermore, making the reflection tool easily accessible and usable to everyone (e.g., in an online version) would allow for gathering, sharing, and comparing data on researchers’ roles across a larger sample, which is imperative to draw more representative conclusions on role patterns. More fundamentally, critical transition and action researchers may want to reframe the role descriptions towards “counter-hegemonic knowledge production” that criticizes “normal” knowledge and experiments with alternative knowledge (Jhagroe 2018, p. 66). Beyond this, we have gathered more specific suggestions for further development, especially of the role survey (see Supplementary Material).

Five major limitations apply to our analysis. First, the designation of natural vs. social scientists was based on current department and disciplinary affiliation, but paints over diverse biographies that, for several people, involve both natural and social science disciplines. To unpack this dimension, future research could investigate links between the biographies and careers of researchers and their roles in ITD projects. Second, the two ITD projects we compared may not be representative for all ITD projects as they were funded by the same funding scheme and have similar science integration designs. Gathering more data about researchers’ roles in ITD projects from other funding contexts and project set-ups may help to substantiate the preliminary patterns we identified. Third, we applied the reflection tool only once in each project. It would be interesting to monitor the change in role profiles in different project phases with varying stakeholder involvement (Stauffacher et al. 2008). Fourth, most researchers in the projects analyzed are affiliated with public research organizations. Role profiles of researchers from other types of organizations, such as independent or private institutes, might look different. Fifth, we focused on interdisciplinary project teams of academic researchers and did not elicit stakeholders’ roles or their perceptions of the roles played by researchers. Gathering such data may be useful for external validation of role profiles.

## Conclusion

The paper set out to explore what is needed to support researchers in making use of the growing literature on the roles they can and do play in ITD projects. We developed and empirically applied a new reflection tool for six key roles of researchers in ITD projects aimed at knowledge co-production, knowledge integration, and solution development. We described its application in two ITD projects in the field of sustainable transformation. We observed that the tool (1) made individual researchers aware of the diverse roles they play; (2) made researchers identify opportunities, challenges, and coping strategies related to this role diversity; (3) revealed differences in their own and others’ perceptions of researchers’ roles within both project teams; (4) depicted the combination of researchers’ roles on project level; and, thereby, (5) sparked project-level discussions about how well this collective role profile aligns with project goals. We conclude that the tool is useful for enhancing reflection on researchers’ roles in ITD projects, noting some aspects for consideration in future application and refinement (see Supplementary Material).

Our contribution can inspire future research into different aspects of roles in ITD research. One aspect is to assess the success of the coping strategies taken up after the reflection workshop as well as their link to more structural changes within academia beyond individual projects (Deutsch et al. 2025b). Moreover, it would be valuable to investigate how the distribution of roles within ITD projects shapes their outputs, notably whether the role diversity we mapped with the reflection tool enhances the ability of researchers to produce robust, salient, and credible knowledge in the eyes of stakeholders (Cash et al. 2003). In transdisciplinary research contexts, the reflection tool could be used to examine how stakeholders view researchers’ role profiles and thus prevent potential misunderstandings due to differences in role perceptions. Another aspect is to see whether reflection in different contexts (e.g., ITD projects with researchers from independent or private institutes) would reveal higher values for action-oriented roles, such as change agent or knowledge broker. Likewise, it might be useful to plan the reflection on roles at the outset of the project to allow for tracing changes over time (cf. Salomon 2023). Researchers could also use ex-ante reflection to indicate which tasks they anticipate to perform in the project and later assess whether they have taken on the anticipated roles. The reflection tool could even be used during the recruitment process of an ITD team to make the role expectations of the project lead and of the candidate transparent. Finally, future research could study career prospects of different roles, especially of undisciplined combinations of knowledge- and process-oriented tasks that may lead researchers into “uncomfortable borderlands between the academy and the larger world” (Robinson 2008, p. 72; cf. Guimarães et al. 2019).

The reflection on roles we proposed is broad and flexible enough to cover and be customized to many different ITD research contexts. On project level, application of the reflection tool to other ITD projects would create comparable data and allow for systematic empirical investigation of researchers’ roles, including the preliminary role patterns we detected with regard to senior and junior researchers as well as natural and social scientists. Additionally, it can complement existing tools aimed at clarifying stakeholder roles in ITD research (e.g., Pohl et al. 2017). At individual level, application of the tool might foster researchers’ awareness of their own roles in different ITD projects and how their roles align. Such increased awareness and clarity of roles is a precondition for harnessing the opportunities and addressing the challenges that many researchers experience in ITD projects aimed at knowledge co-production, knowledge integration, and solution development.

## Supplementary Information

Below is the link to the electronic supplementary material.

Supplementary file1 (DOCX 1342 kb)

## Data Availability

The data that support the findings of this study are not openly available due to reasons of sensitivity and are available from the corresponding author upon reasonable request. Data are located in controlled access data storage at Eawag.

## Footnotes

1 The role reflection tool was developed by Benjamin Hofmann and Sabine Hoffmann (with comments by Rea Pärli) and used by Hanna Salomon, amongst others, during her master’s thesis. Parts of this paper are based on findings presented in Hanna Salomon’s master’s thesis.

2 An exception are two team members in TRAPEGO from an independent research institute. For more information on both projects, see www.trapego.ch/en and https://narp.ethz.ch/en/research/ongoing-projects/trebridge.html

3 Data from researchers who could not attend the workshop was gathered electronically shortly after the workshop. In TRAPEGO, this was the case for four researchers, in TREBRIDGE for two. The paper authors themselves are part of the data: BH in TRAPEGO, HS in TREBRIDGE, and SH in both TRAPEGO and TREBRIDGE.
