# Supplementary material for: Roles of researchers in inter- and transdisciplinary sustainability research: a reflection tool
Source: Sustain Sci. 2025 Feb 17;20(3):777–92. doi: 10.1007/s11625-024-01619-x (PMC12033213; doi:10.1007/s11625-024-01619-x)
Supplement: Supplementary file 1 — Supplementary file1 (DOCX 1342 kb) [file 11625_2024_1619_MOESM1_ESM.docx]

Supplementary Material

Benjamin Hofmann^[[1]](#footnote-1)^, Hanna Salomon^1,^^[[2]](#footnote-2)^, Sabine Hoffmann^1,^^[[3]](#footnote-3)^

Contents

[Sample Role Workshop Program 2](#_Toc174095976)

[Role survey used in the workshops 3](#_Toc174095977)

[Spider webs developed during the workshops: TRAPEGO 6](#_Toc174095978)

[Breakout group 1 6](#_Toc174095979)

[Breakout group 2 8](#_Toc174095980)

[Breakout group 3 10](#_Toc174095981)

[Spider webs developed during the workshops: TREBRIDGE 12](#_Toc174095982)

[Breakout group 1 12](#_Toc174095983)

[Breakout group 2 14](#_Toc174095984)

[Breakout group 3 16](#_Toc174095985)

[Future improvement and application 17](#_Toc174095986)

[References 18](#_Toc174095987)

# Sample Role Workshop Program

| **Introduction**   - Present literature on researchers’ roles - Introduce typology of roles - Explain individual and group exercise   **Individual Exercise**   - People fill out a short role survey on paper that produces a score for each role - People map the score for each role on an individual spider web, and then copy it to a breakout group spider web poster - People reflect on opportunities and challenges they might face with respect to these roles (e.g., tensions, resources, expectations) and coping strategies they might develop   **Breakout groups**   - People briefly present the various actual or anticipated roles they assume and share the challenges and opportunities they might face as well as the coping strategies they might develop - Moderators note challenges and opportunities on flip chart |
| --- |
| **Plenary discussion**   - Moderators briefly summarize the breakout group work results with a focus on the role profile of the breakout group and the main opportunities, challenges, and coping strategies identified - Entire group discusses the question: “To what extent does the combination of roles in our team fit the project goals?” - Group agrees on implications of the discussion and next steps, e.g., need for adaptation in role distribution, repeated application of the tool at later point in time, etc. |

# Role survey used in the workshops^[[4]](#footnote-4)^

[Name of specific ITD project]: Discussion on our various roles as scientists

**YOUR NAME: _____________________**

1. **Please fill in the following questions and sum up the scores for each role**

| **1) Traditional scientist (reflective scientist)** | **YES 🡪 put 1**  **NO 🡪 put 0** |
| --- | --- |
| **Within [name of specific ITD project], I engage in the following activity** |  |
| Deconstruct complex problems into solvable parts (e.g., by applying scientific concepts and theories) |  |
|  |  |
| Conduct or supervise the conduct of systematic analyses of deconstructed problems and potential solutions (e.g., by using quantitative and/or qualitative methods) |  |
|  |  |
| Communicate scientific knowledge validated as intersubjective by the respective discipline |  |
|  |  |
| **SUM *(map this score in spider web)*** |  |

| **2) Self-reflexive scientist** | **YES 🡪 put 1**  **NO 🡪 put 0** |
| --- | --- |
| **Within [name of specific ITD project], I engage in the following activity** |  |
| Observe and reflect on research practices (e.g., by writing observation protocols or research diaries) |  |
|  |  |
| Critically reflect on internal and external power dynamics that shape the project (e.g., by identifying hierarchies, differences in resource endowment) |  |
|  |  |
| Critically reflect on own normative orientation in relation to project goals (e.g., by reflecting on own personal motivations, attitudes, and policy preferences) |  |
|  |  |
| **SUM *(map this score in spider web)*** |  |

| **3) Knowledge integrator** | **YES 🡪 put 1**  **NO 🡪 put 0** |
| --- | --- |
| **Within [name of specific ITD project], I engage in the following activity** |  |
| Cross or bridge boundaries of different disciplines or fields (e.g., by linking theoretical concepts from different disciplines or fields, co-creating integrative frameworks, or developing interdisciplinary methods) |  |
|  |  |
| Synthesize knowledge from different disciplines or fields and generate new integrated knowledge (e.g., by recognizing critical connections and leveraging potential synergies) |  |
|  |  |
| Design, plan, monitor, assess, and evaluate integrative processes and their integrated outputs |  |
|  |  |
| **SUM *(map this score in spider web)*** |  |

| **4) Knowledge broker** | **YES 🡪 put 1**  **NO 🡪 put 0** |
| --- | --- |
| **Within [name of specific ITD project], I engage in the following activity** |  |
| Identify and connect relevant actors from science, policy, practice, and/or the public (e.g., understanding the actor landscape, identifying and mediating different perspectives) |  |
|  |  |
| Bridge different types of knowledge (e.g., by making scientific knowledge accessible for different target audiences and/or integrating knowledge from policy, practice, and/or the public into the scientific process) |  |
|  |  |
| Translate, interpret, adapt, and tailor different types of knowledge to different target audiences (e.g., by finding realistic problem-solution couplings) |  |
|  |  |
| **SUM *(map this score in spider web*)** |  |

| **5) Process facilitator** | **YES 🡪 put 1**  **NO 🡪 put 0** |
| --- | --- |
| **Within [name of specific ITD project], I engage in the following activity** |  |
| Initiate and facilitate learning processes or experiments within project team and/or with actors from policy, practice, and/or the public |  |
|  |  |
| Organize and prepare workshops, select and invite actors (from science, policy, practice, and/or the public), and condense the outcomes |  |
|  |  |
| Provide space for critical reflection and deliberation (e.g., by encouraging expression of different viewpoints) |  |
|  |  |
| **SUM *(map this score in spider web)*** |  |

| **6) Change agent** | **YES 🡪 put 1**  **NO 🡪 put 0** |
| --- | --- |
| **Within [name of specific ITD project], I engage in the following activity** |  |
| Strategically network with actors from science, policy, practice, and/or the public in the context of change processes (e.g., by engaging with actors’ concerns and generating possible solutions for real-world problems) |  |
|  |  |
| Intervene into policy, practice, and/or the public with the aim to contribute to change (e.g., via policy consultations, innovation processes in relation to practices, and reconfiguration of actor relations) |  |
|  |  |
| Empower actors from policy, practice, and/or the public to lead own change processes (e.g., through motivation and capacity-building) |  |
|  |  |
| **SUM *(map this score in spider web)*** |  |

1. **Map your score for each role in the spider web**

**
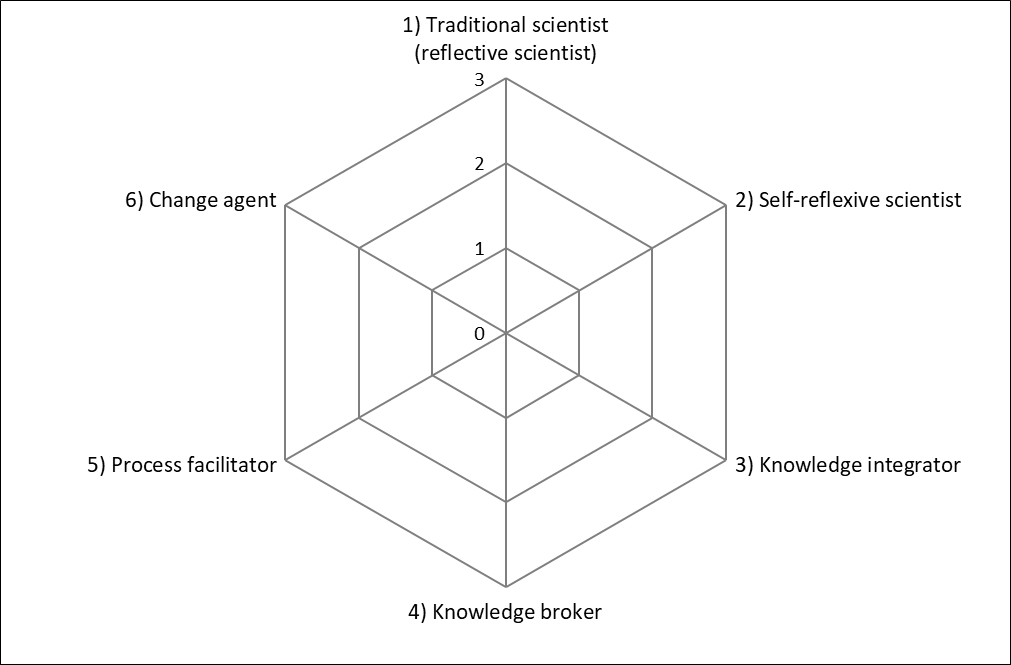
**

1. **Copy your spider web to the poster of your breakout group**
2. **Reflect on opportunities and challenges you anticipate with respect to these roles (e.g., tensions, resources, expectations) and coping strategies you/we might develop**

# Spider webs developed during the workshops: TRAPEGO

## Breakout group 1


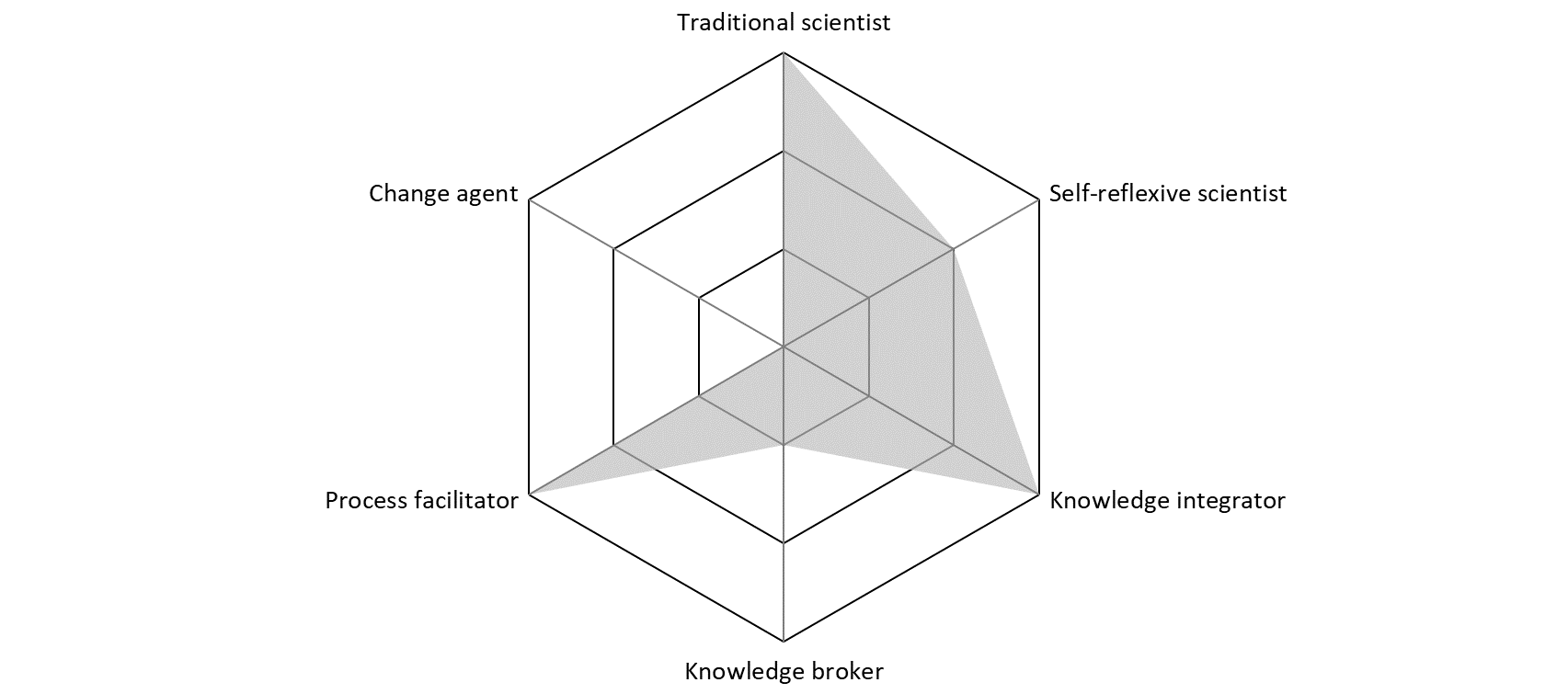
Researcher A.1


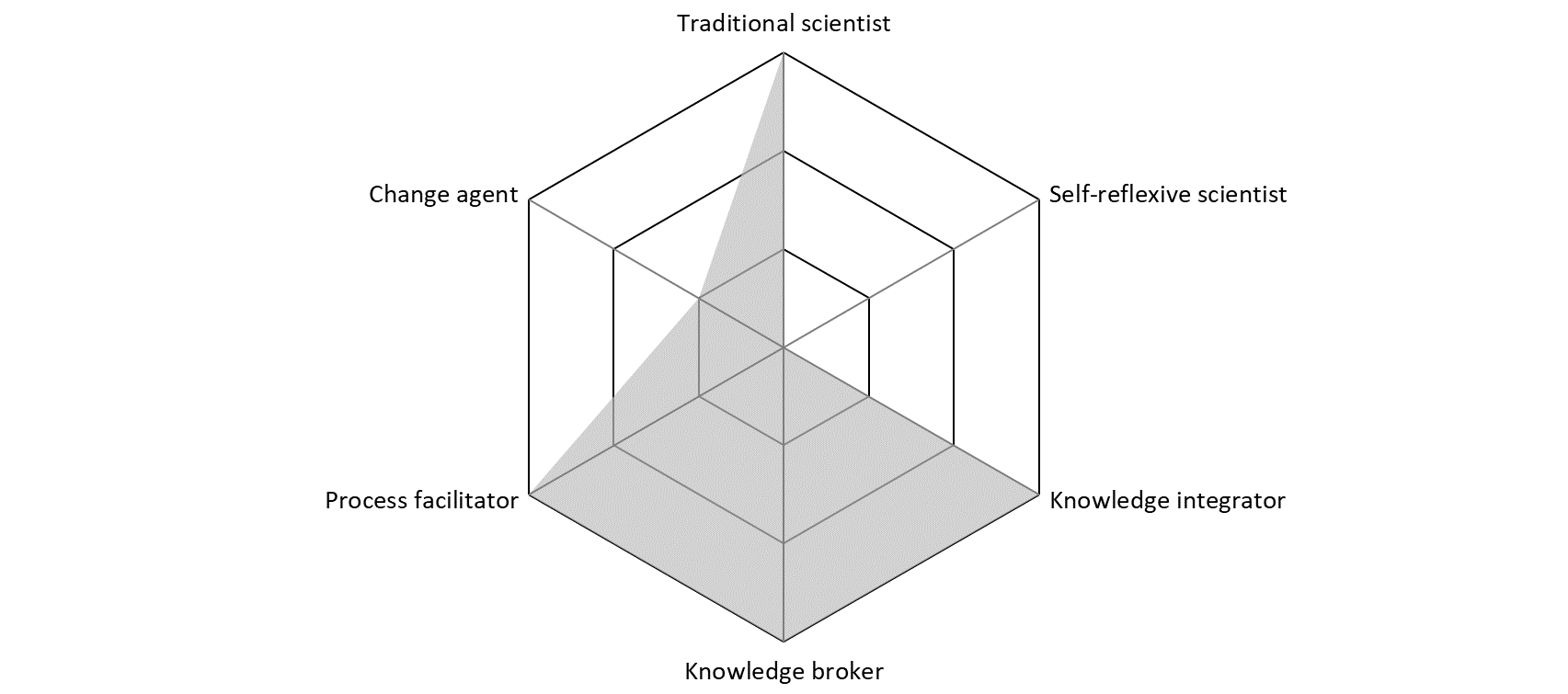
Researcher A.2


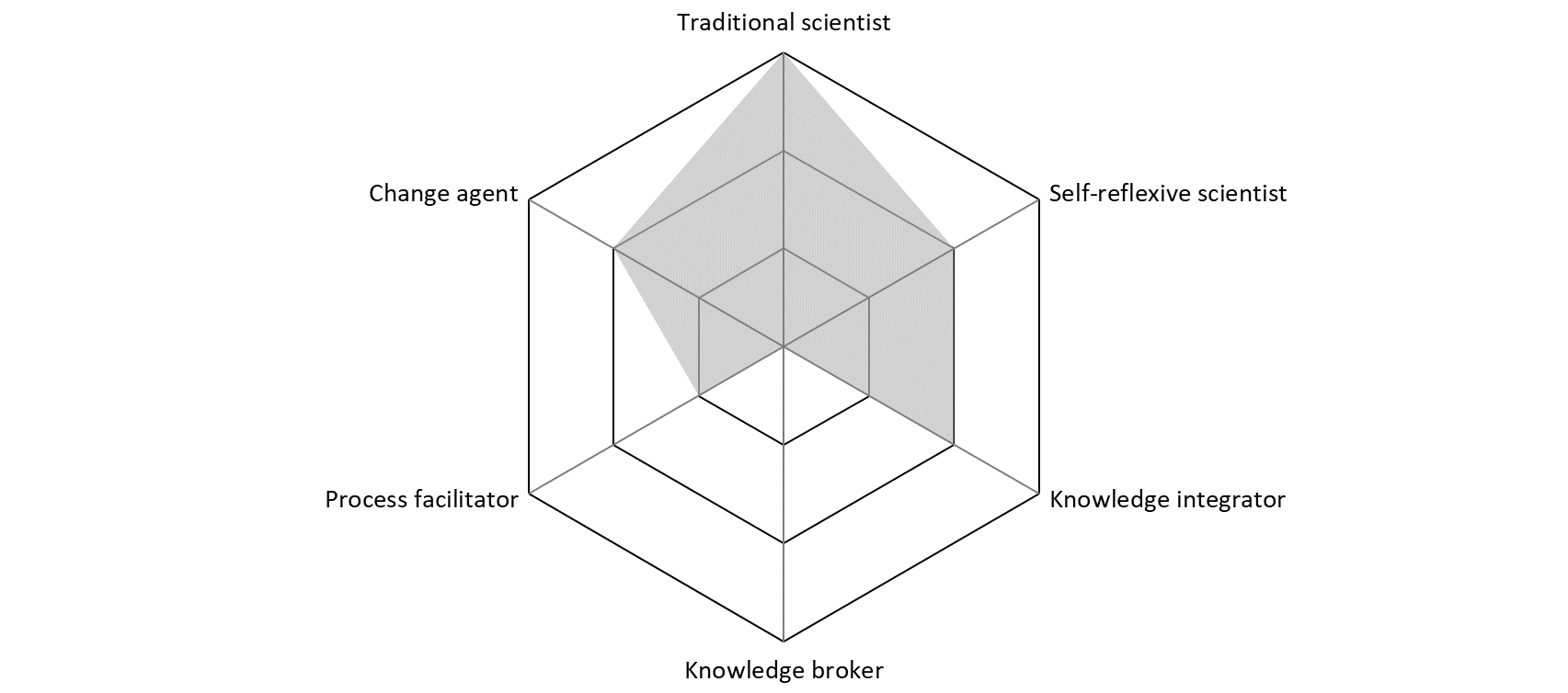
Researcher A.3


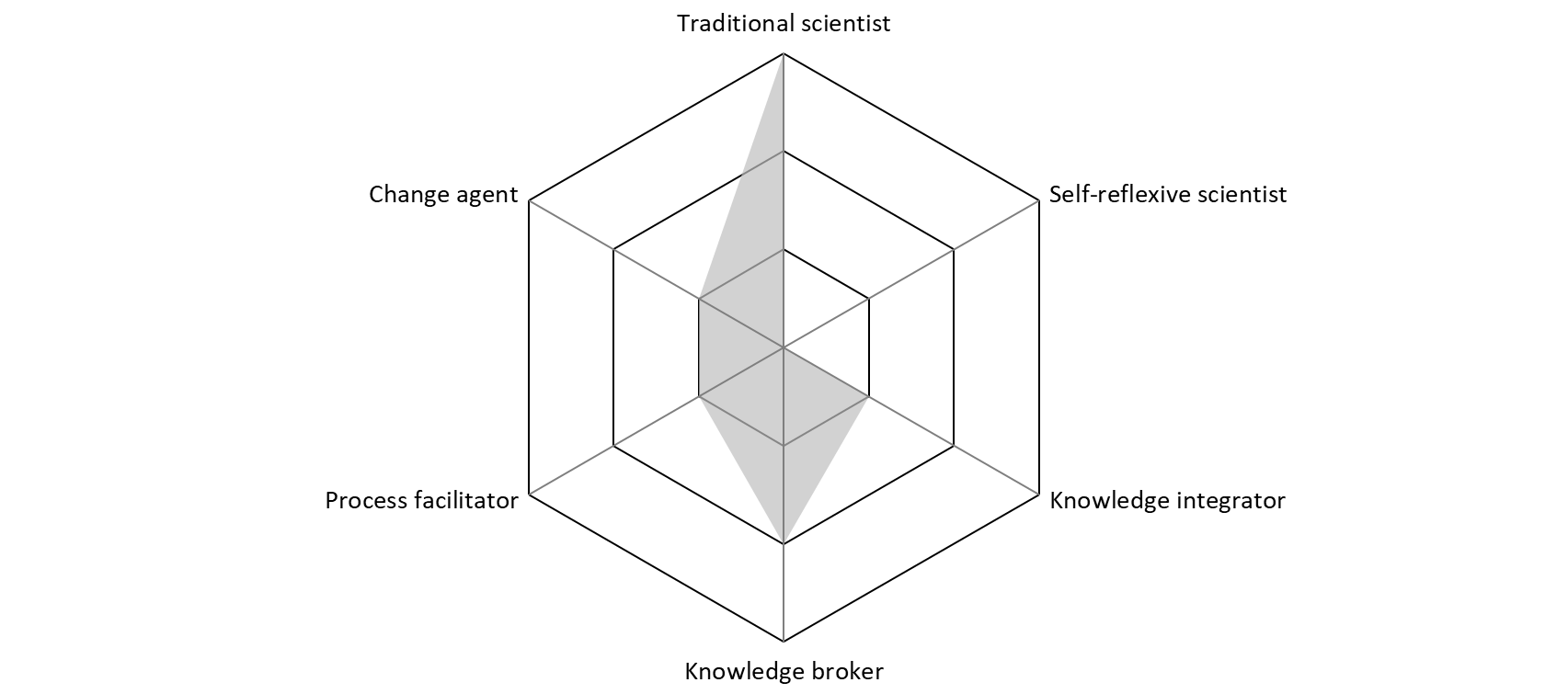
Researcher A.4


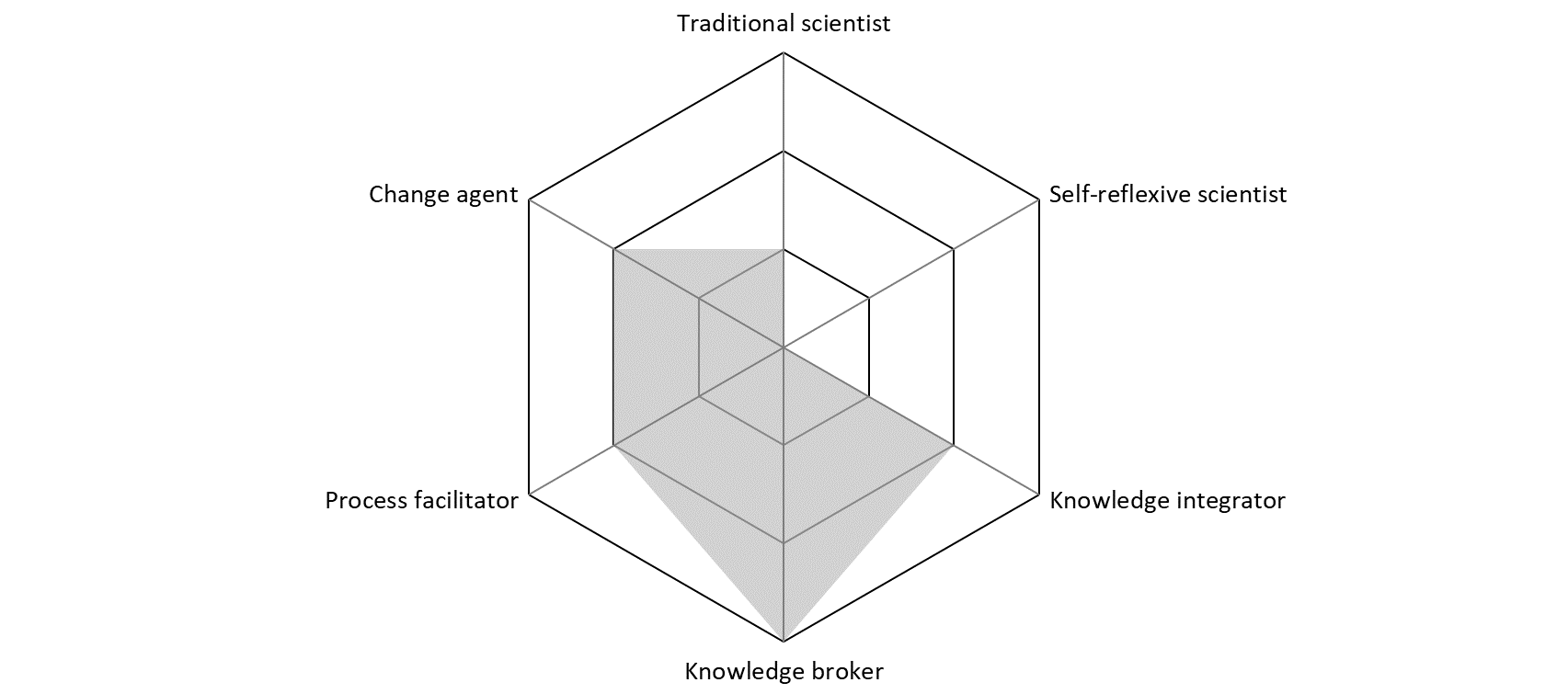
Researcher A.5


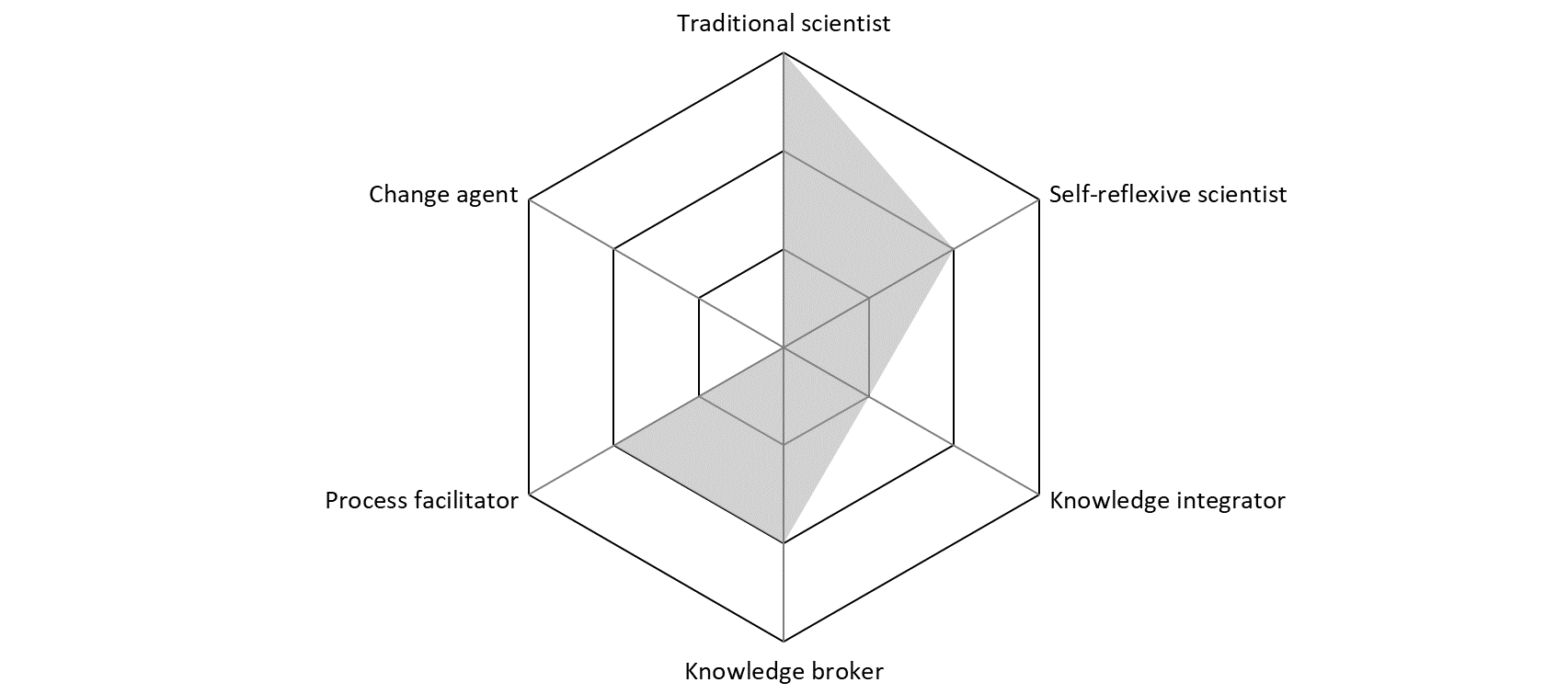
Researcher A.6

## Breakout group 2


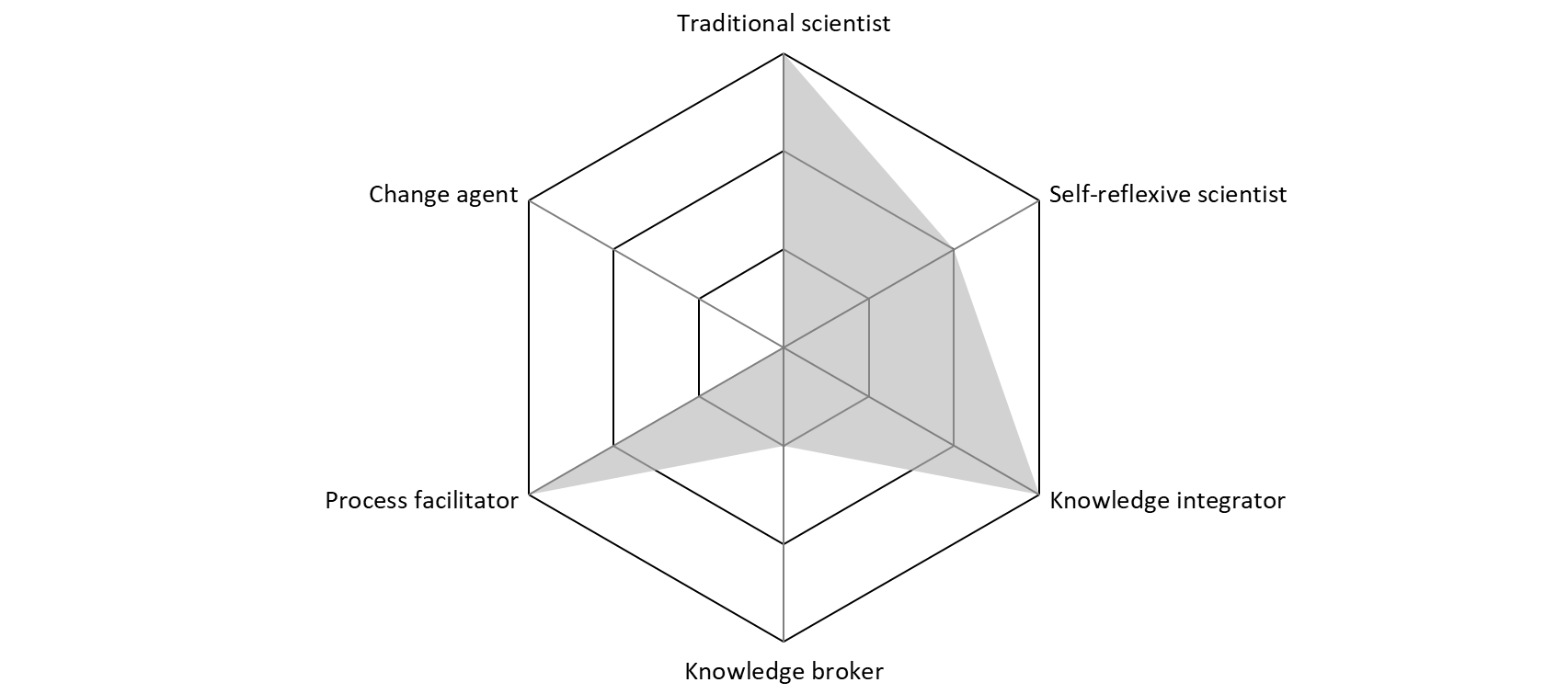
Researcher A.7


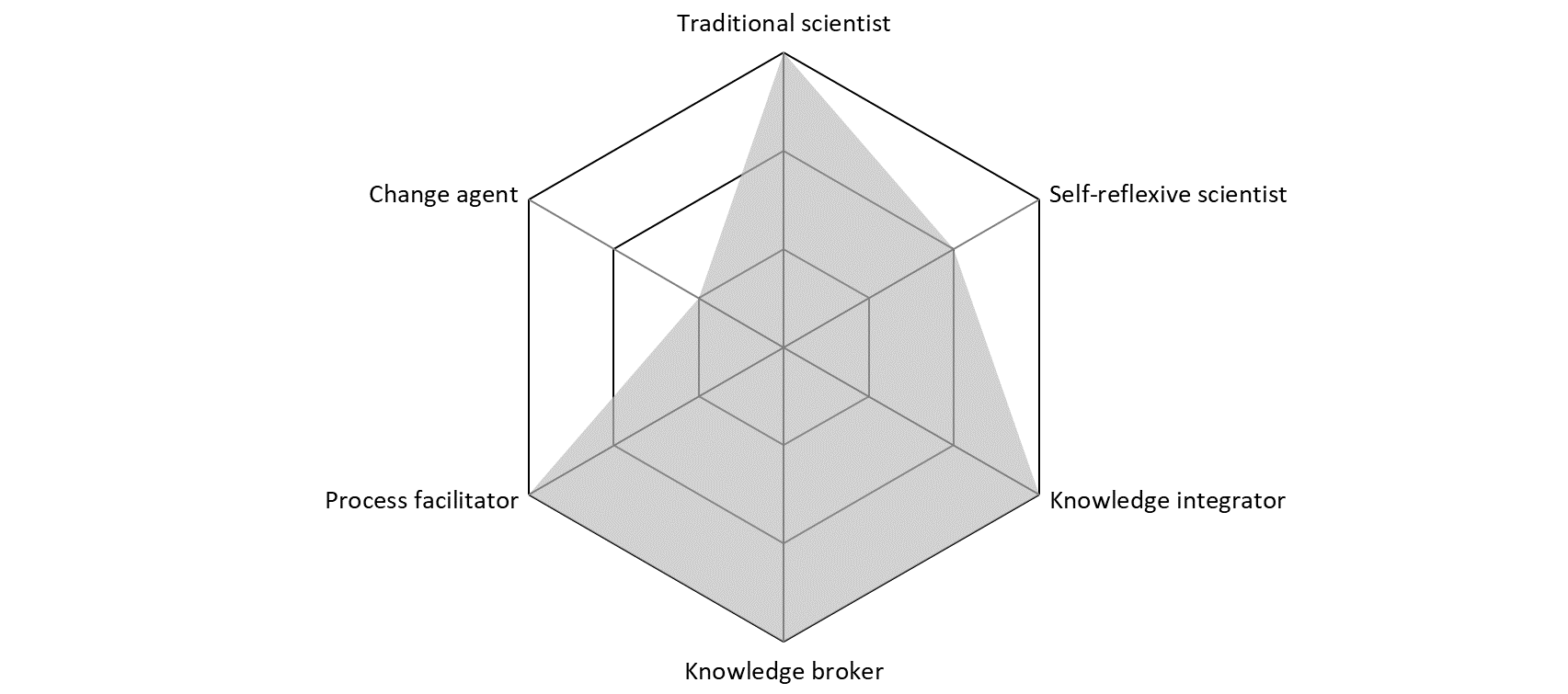
Researcher A.8


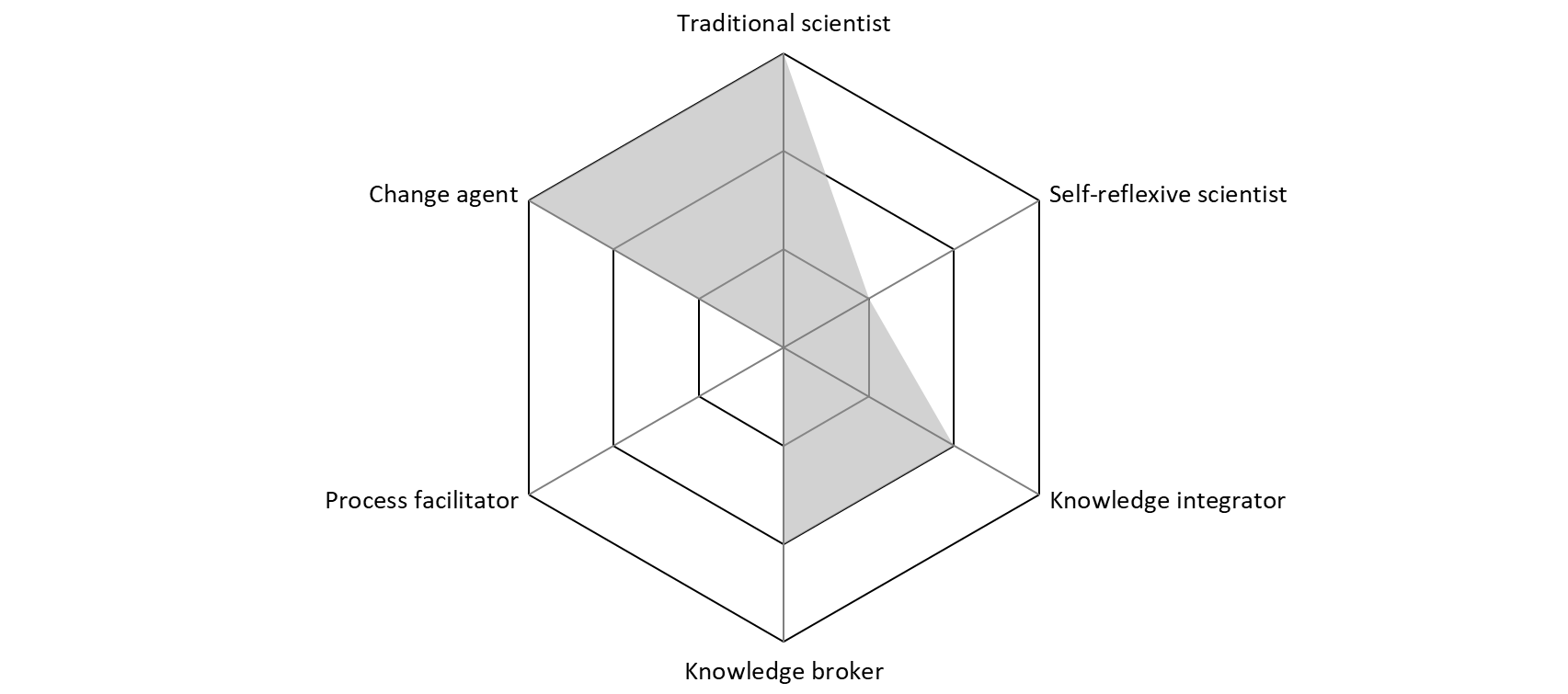
Researcher A.9


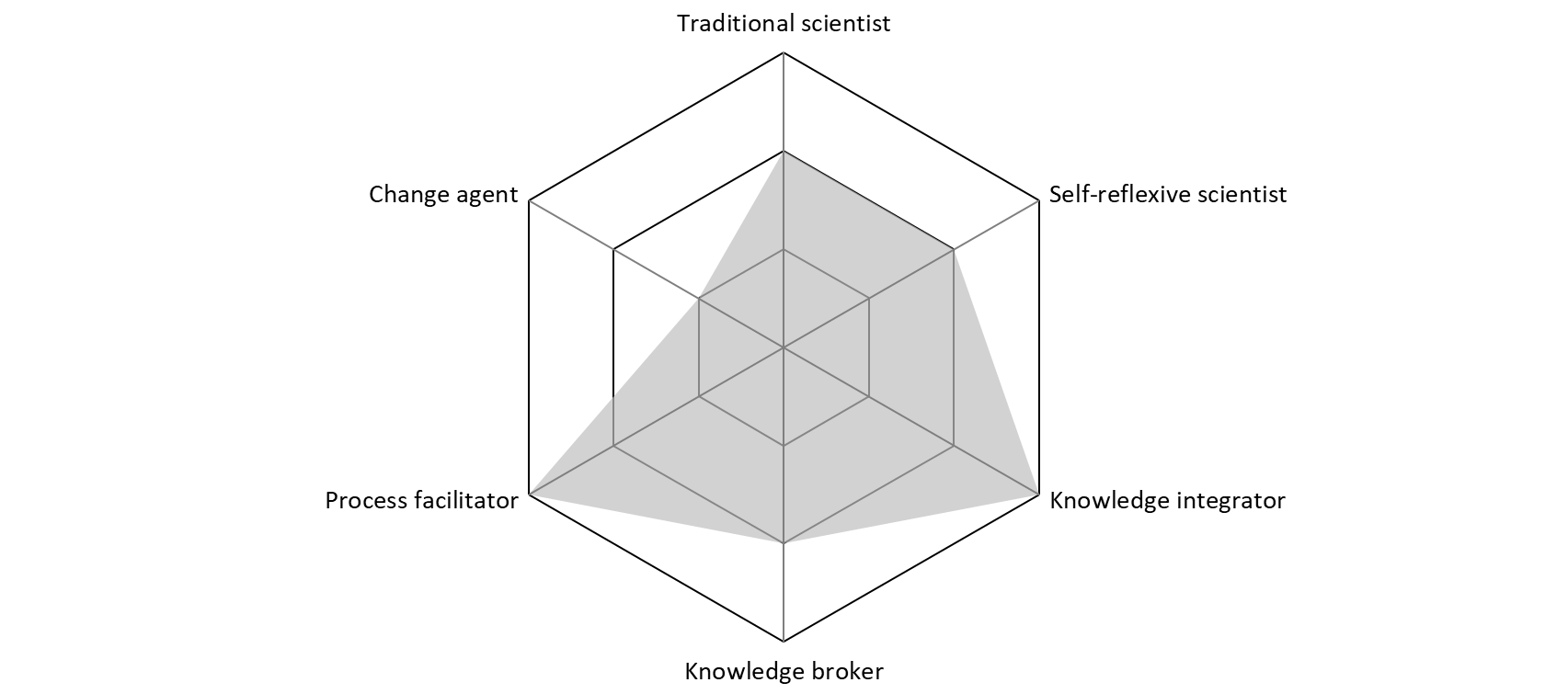
Researcher A.10


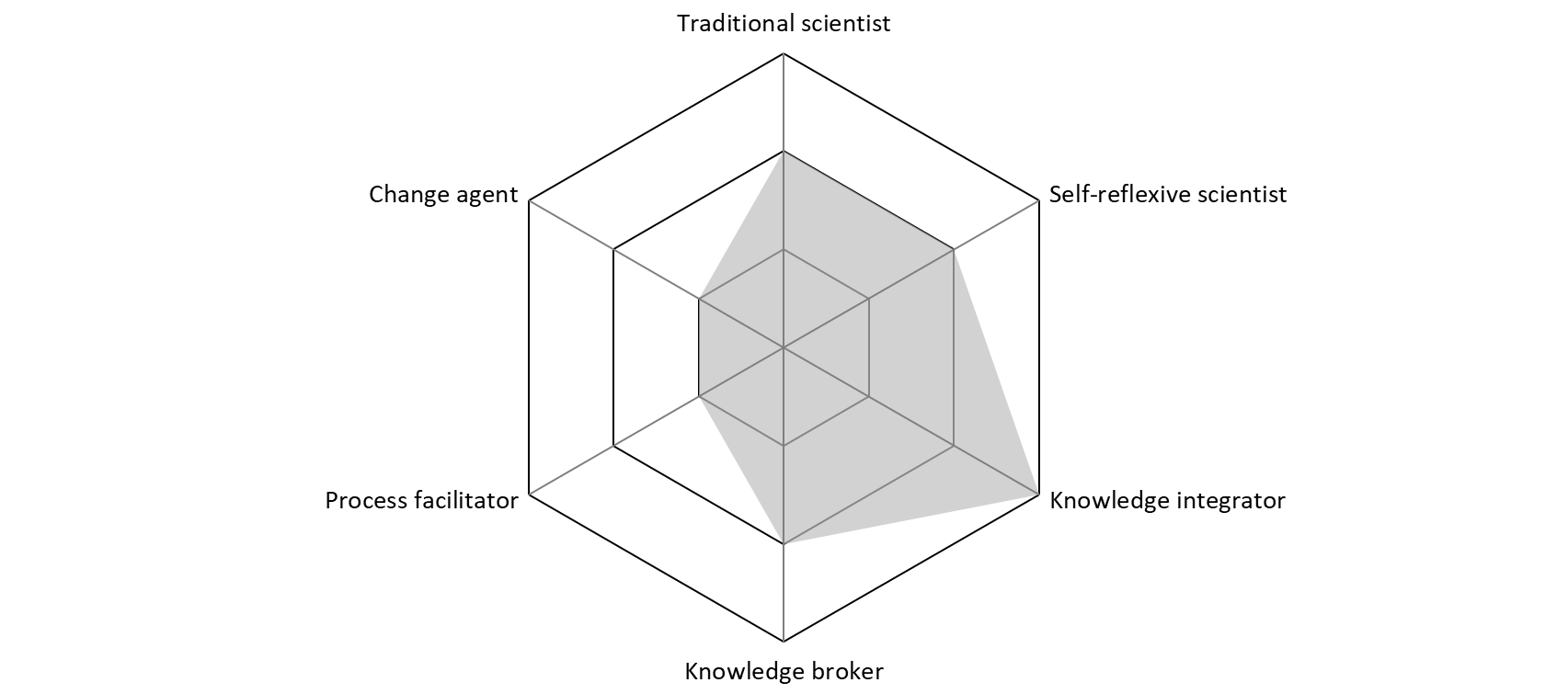
Researcher A.11

## Group 3

*(collected after workshop, not part of group discussion)*


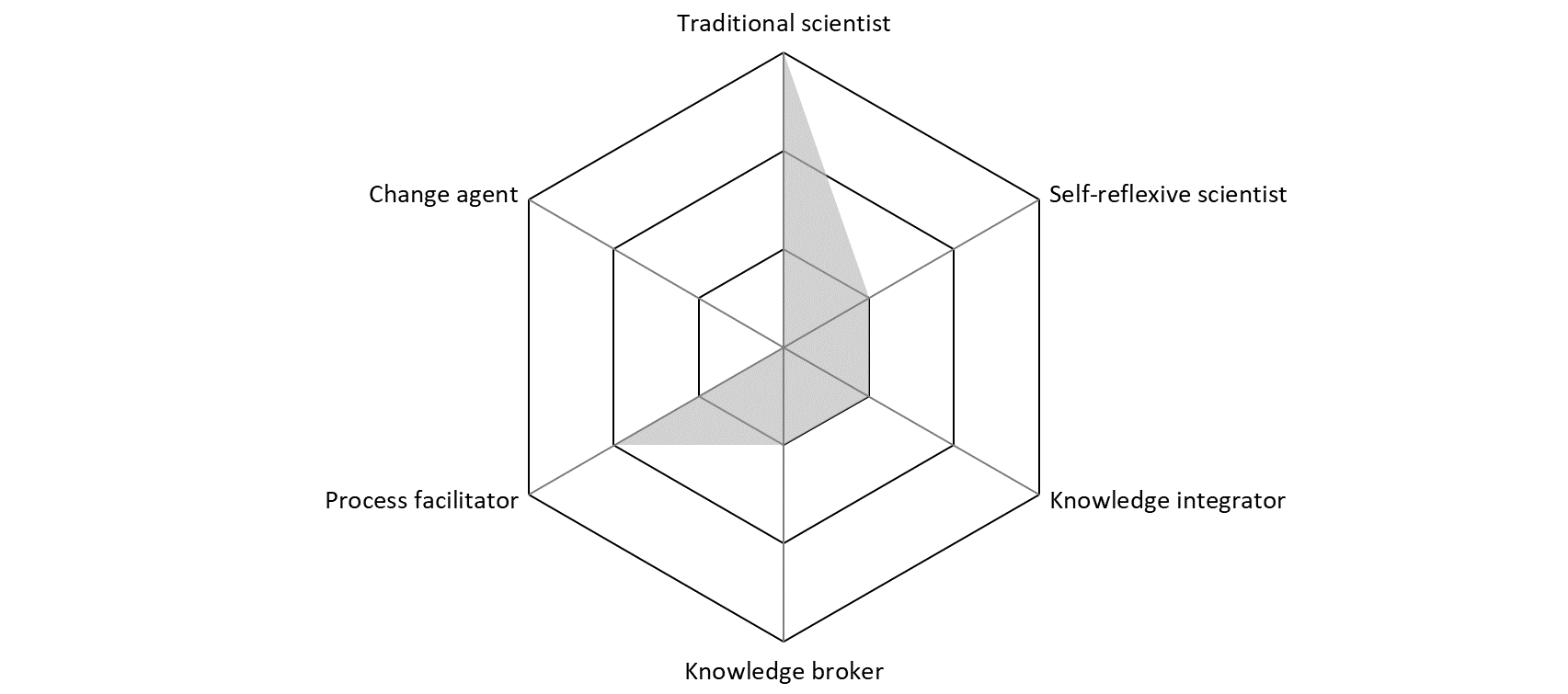
Researcher A.12


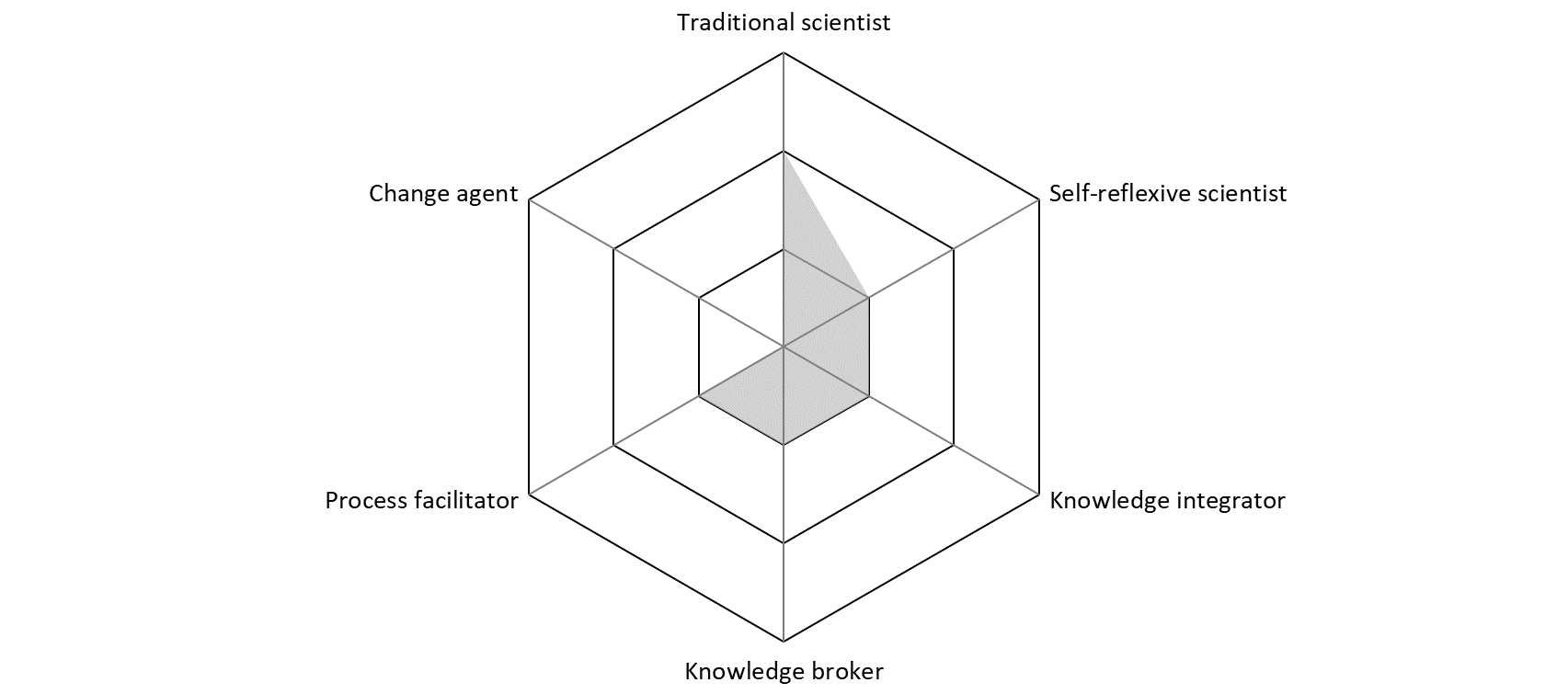
Researcher A.13


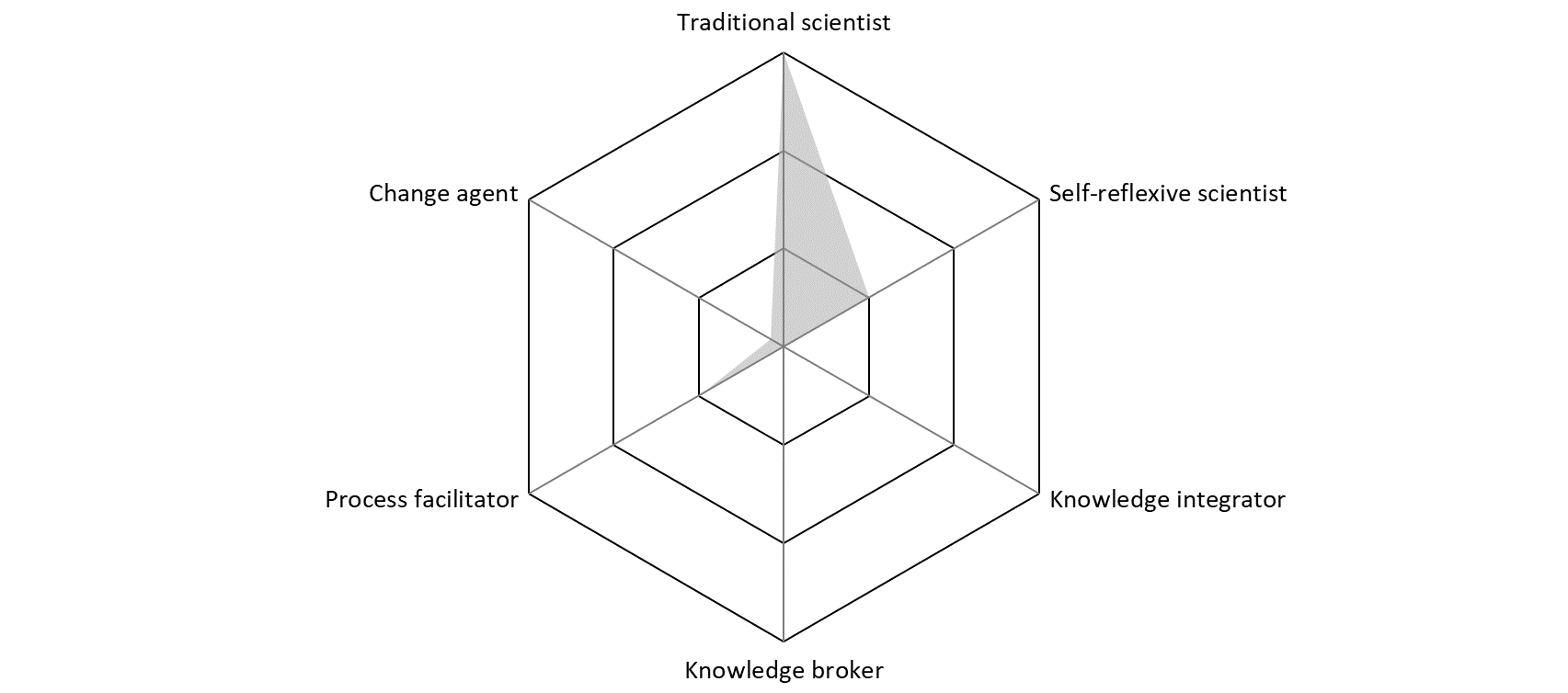
Researcher A.14

Researcher A.15


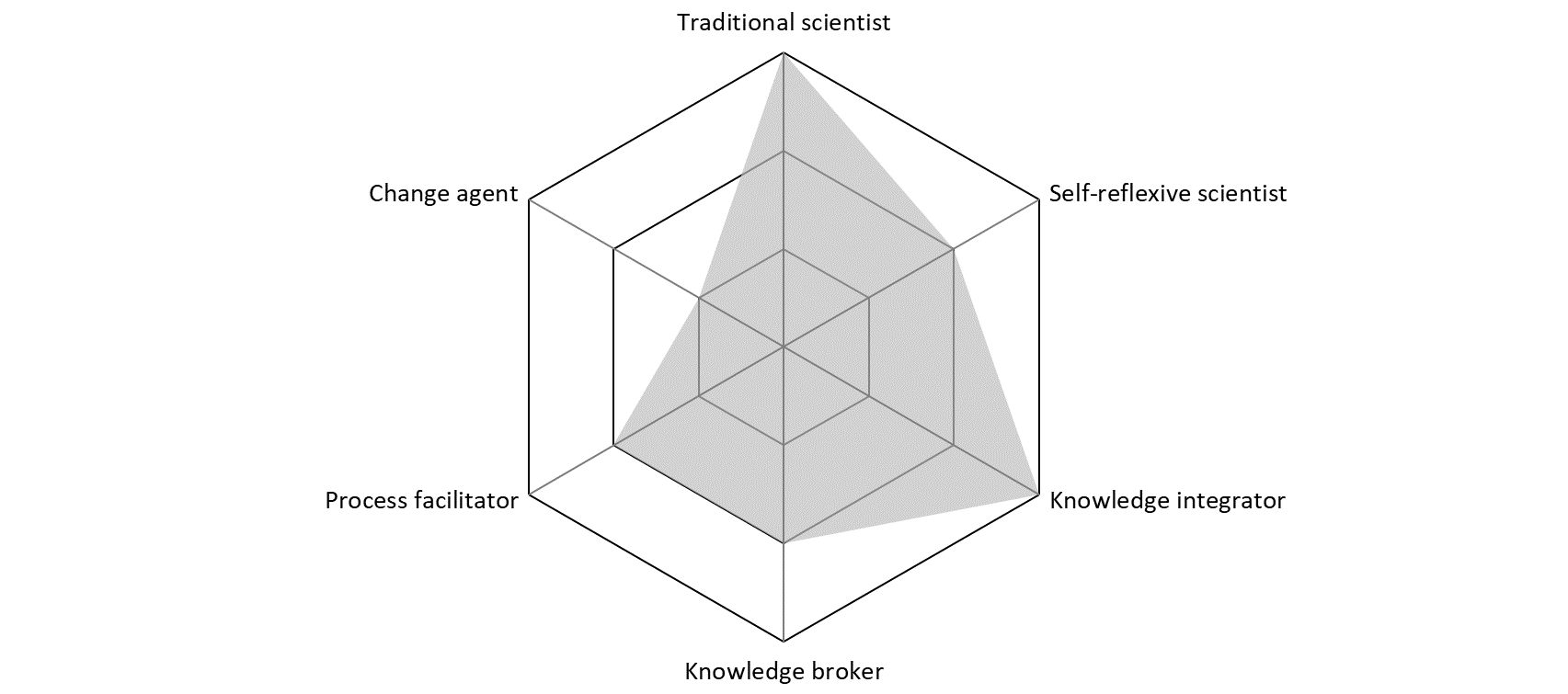

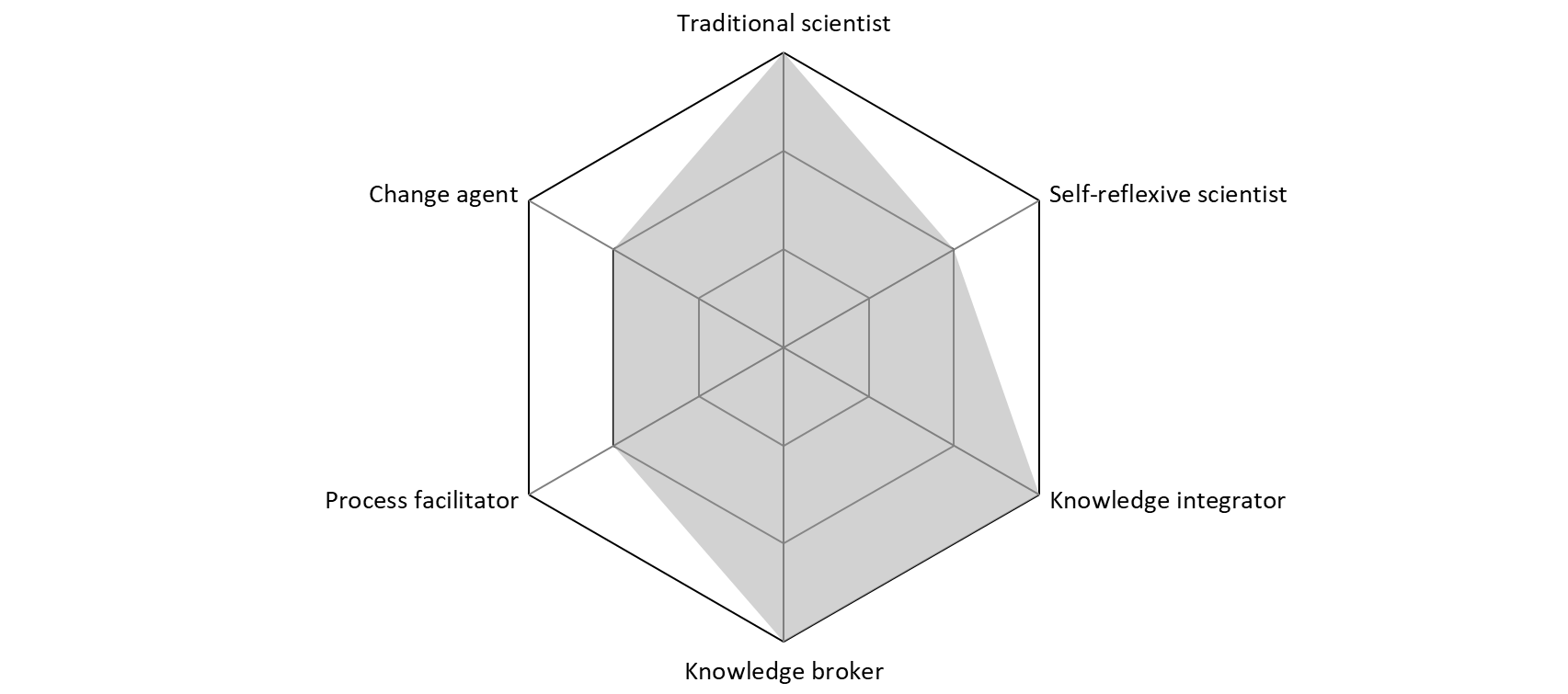
Researcher A.16

# Spider webs developed during the workshops: TREBRIDGE

## Breakout group 1

Researcher B.1


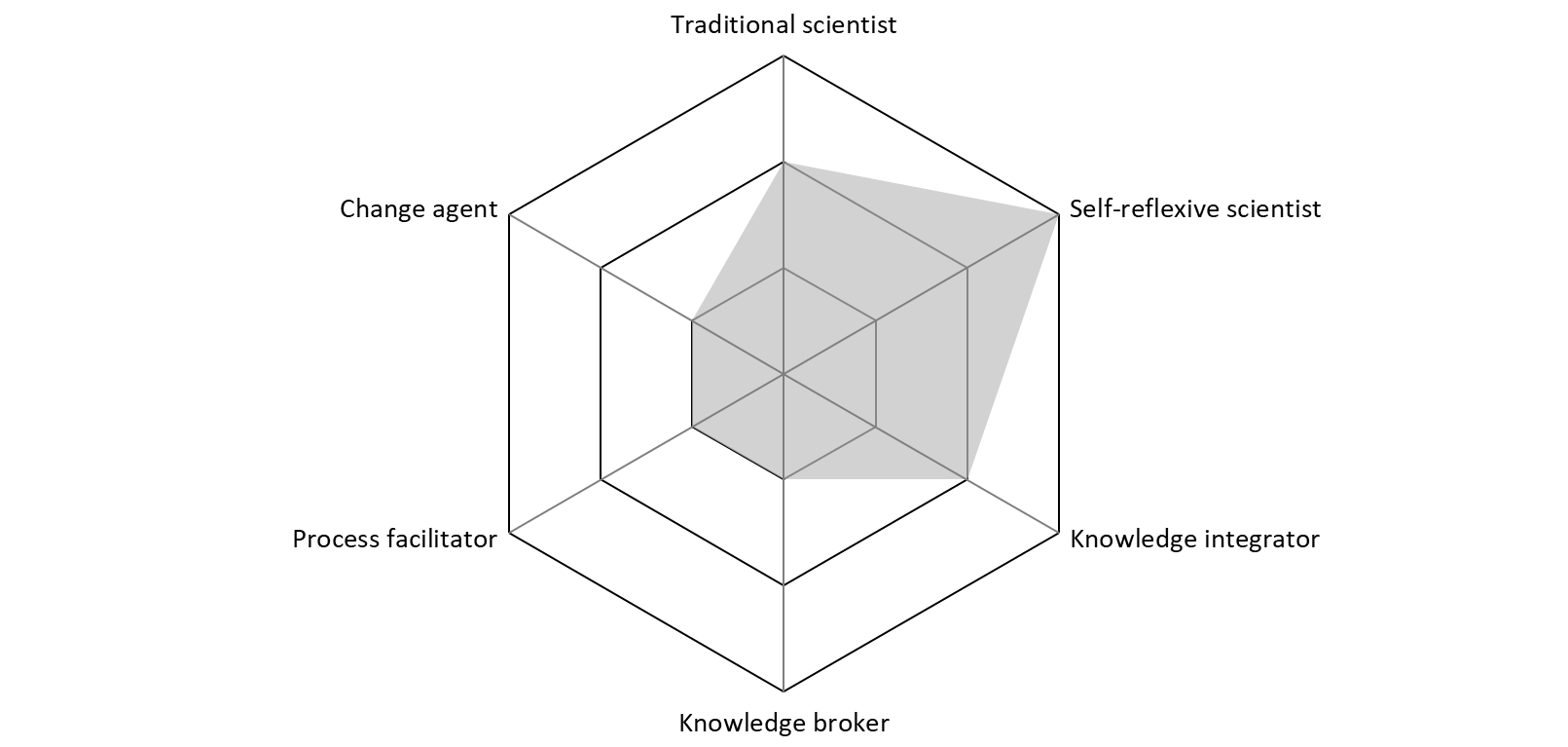


Researcher B.2


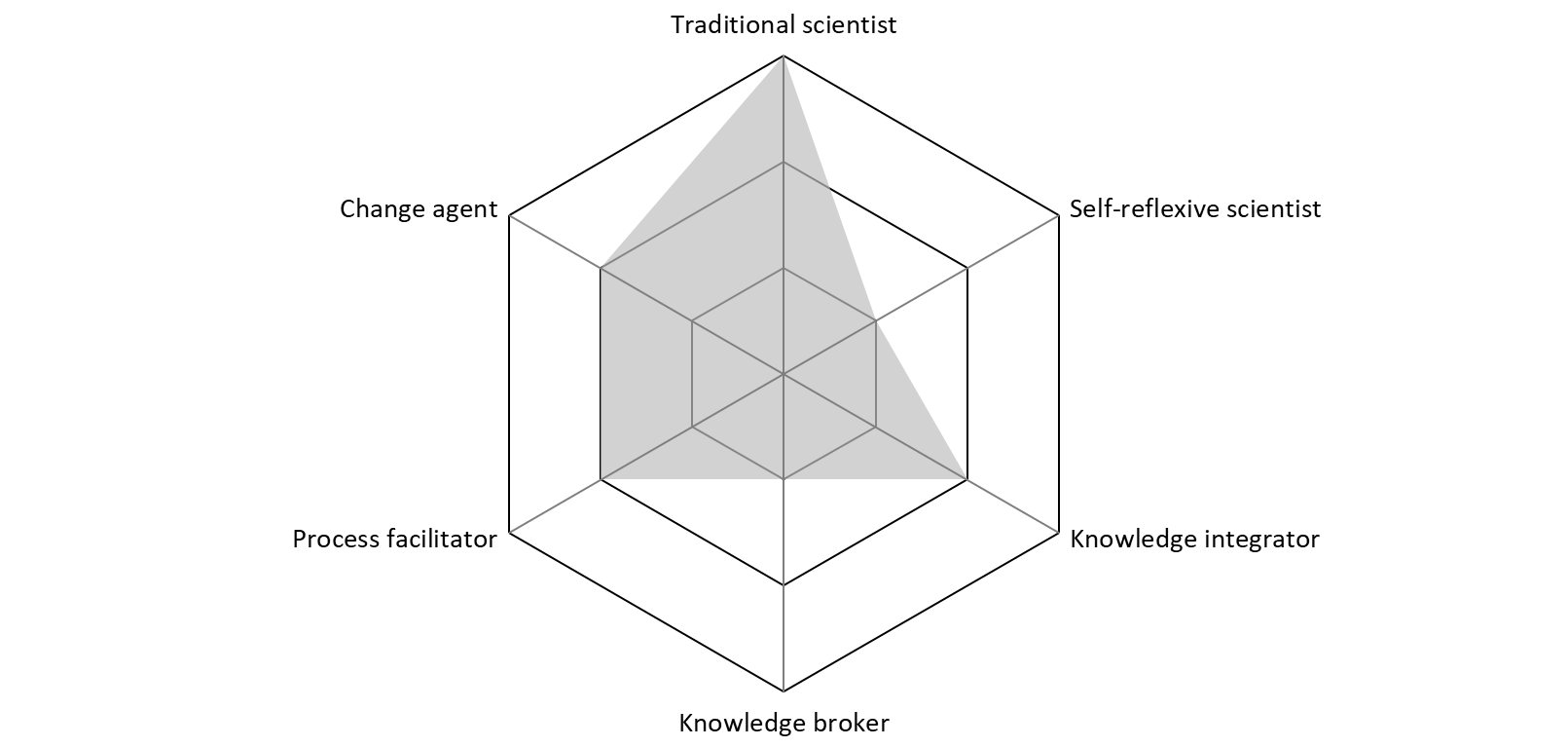

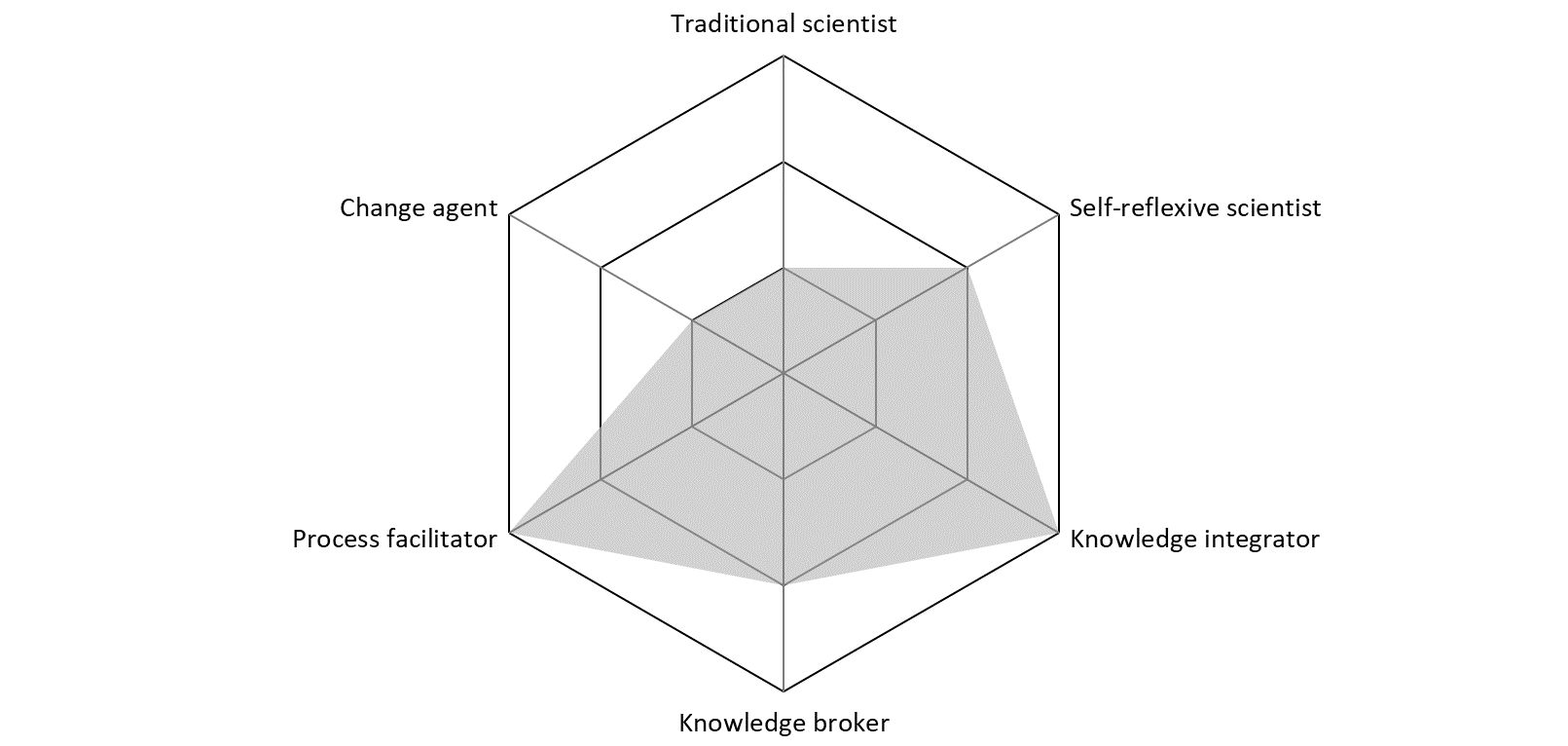
Researcher B.3

Researcher B.4


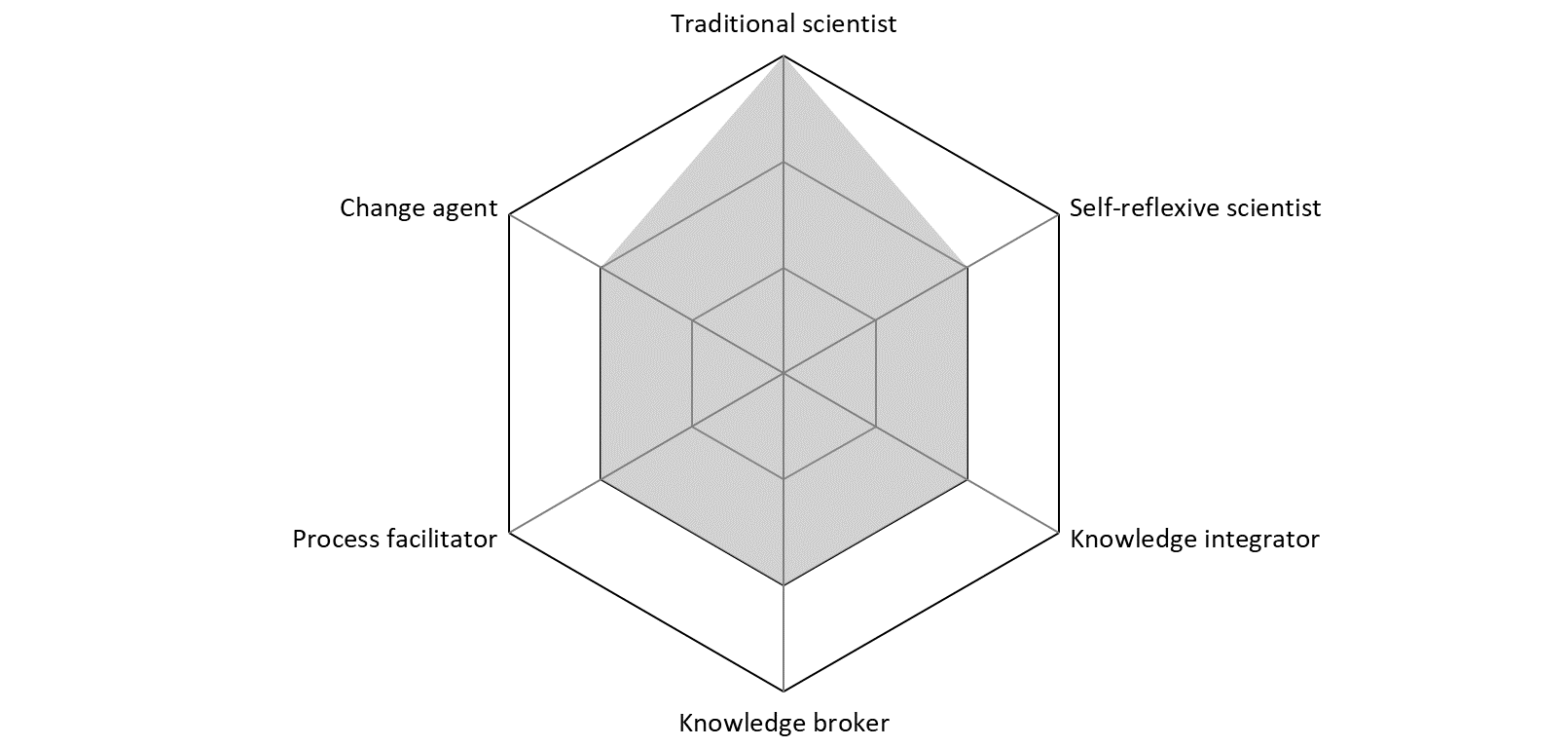
Researcher B.5


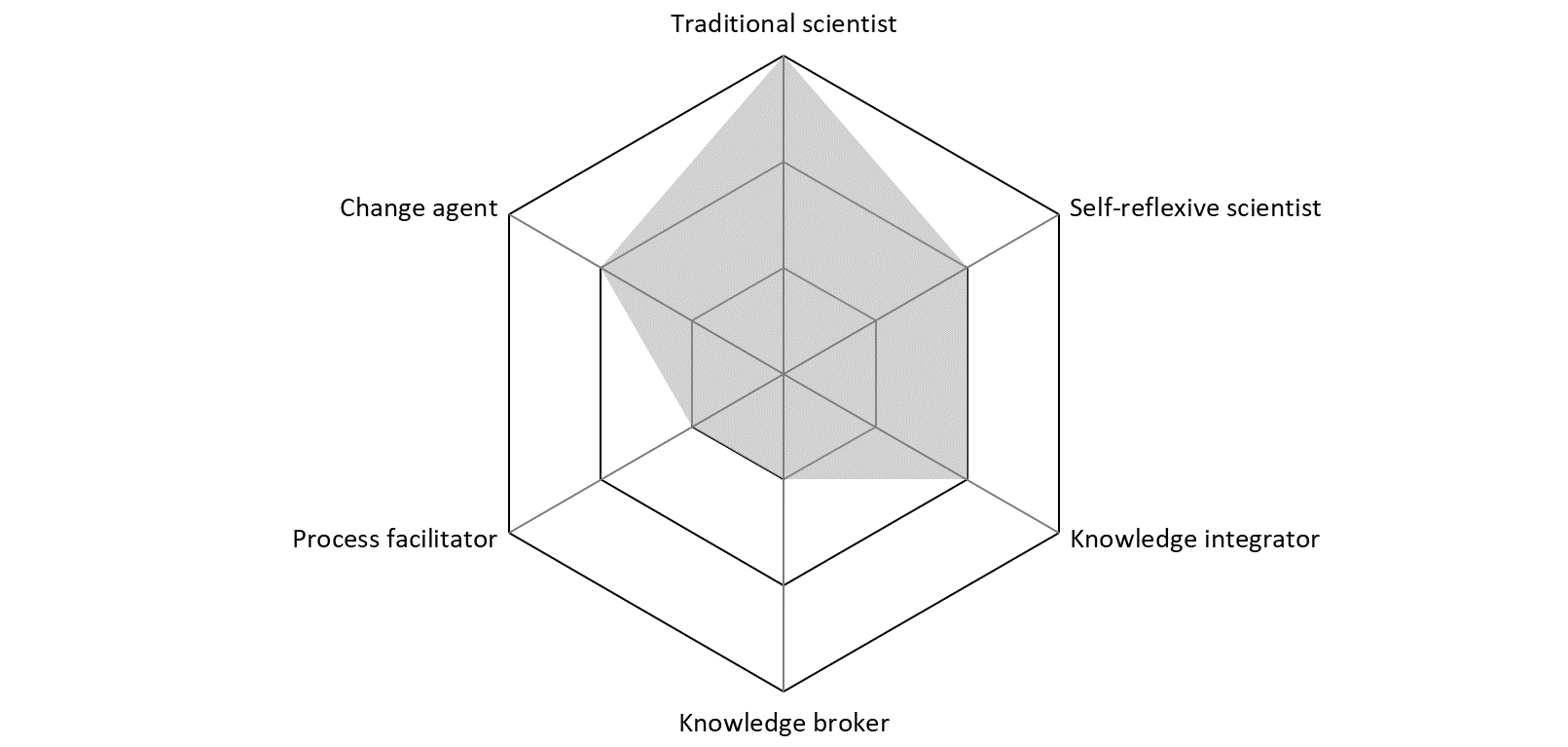


Researcher B.6


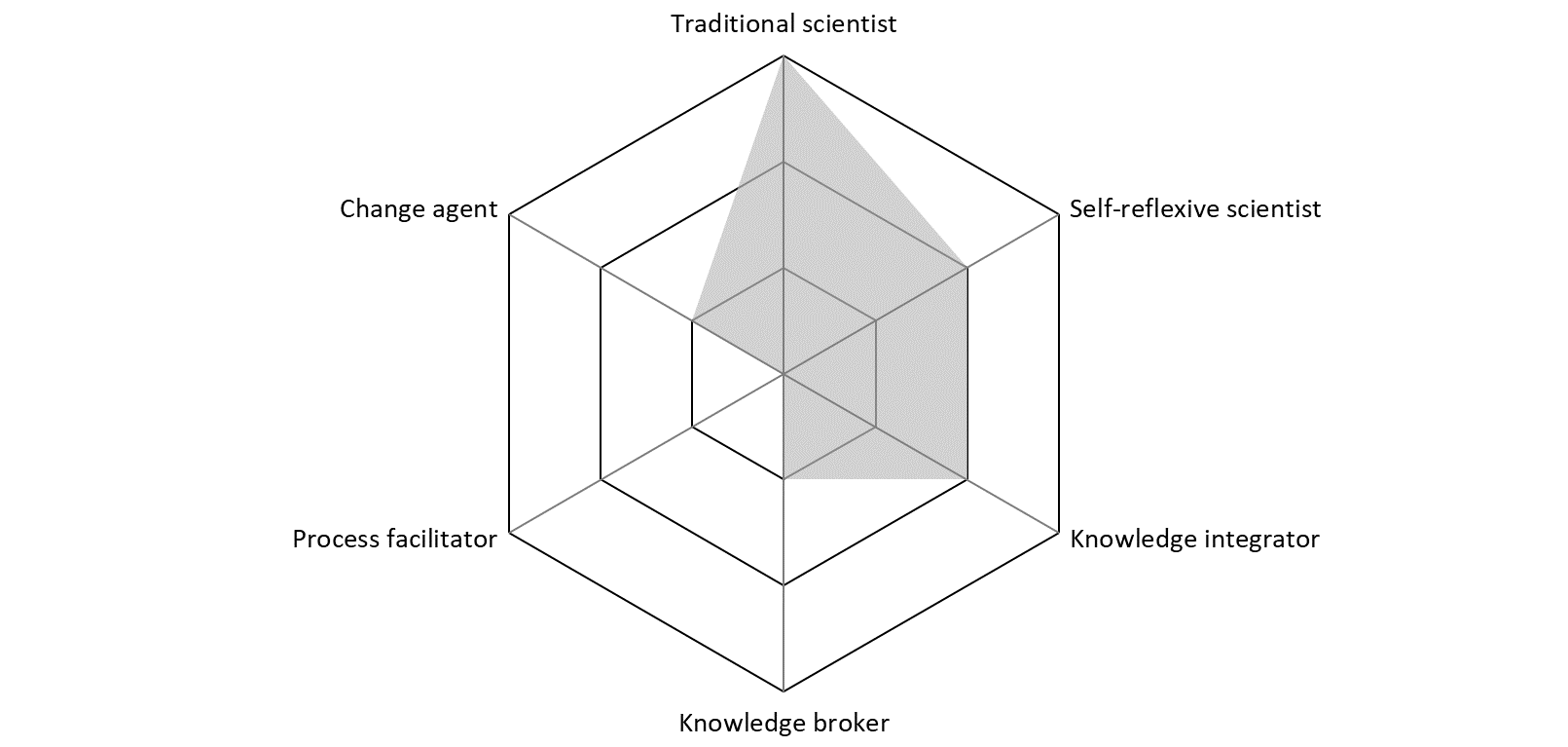


## Breakout group 2

Researcher B.7


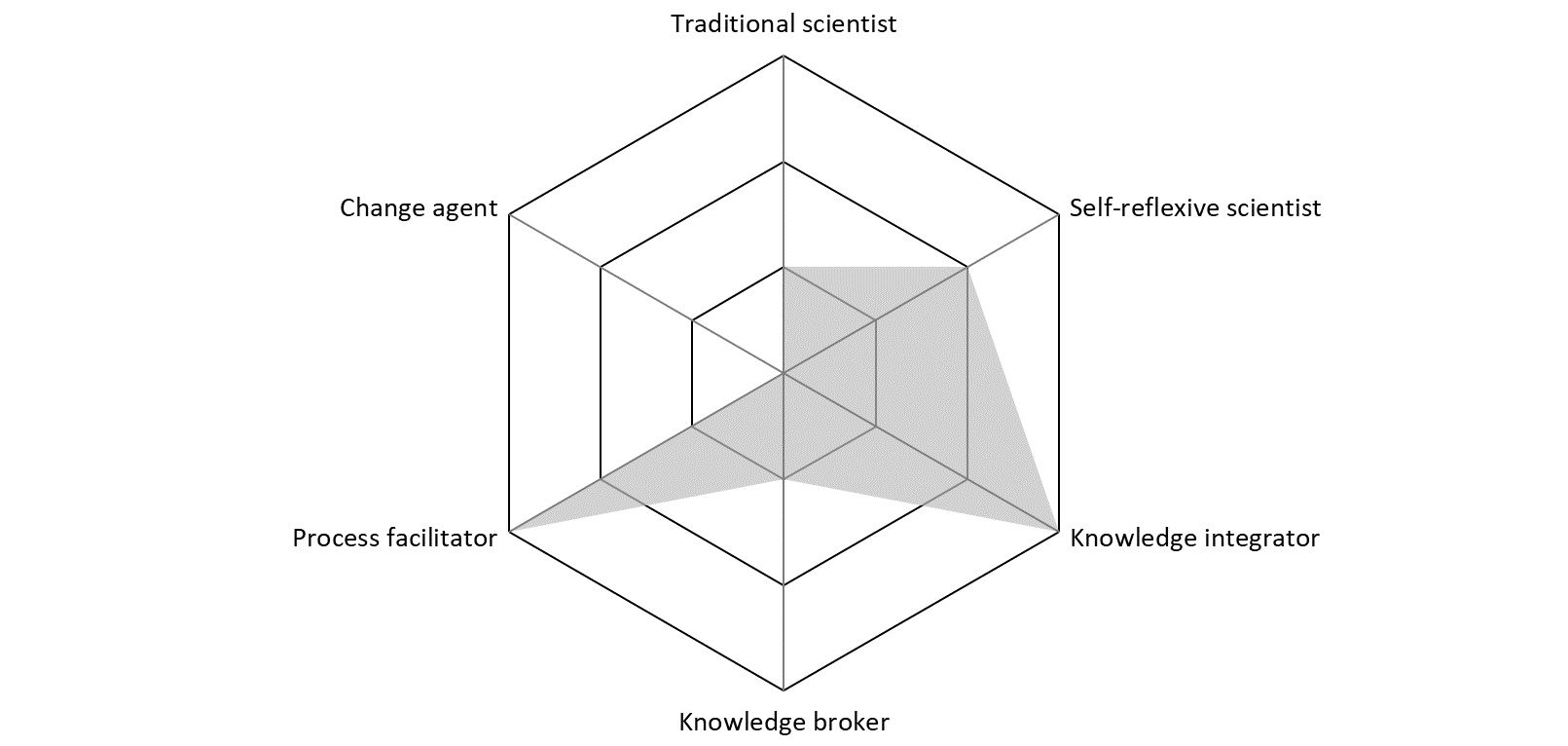


Researcher B.8
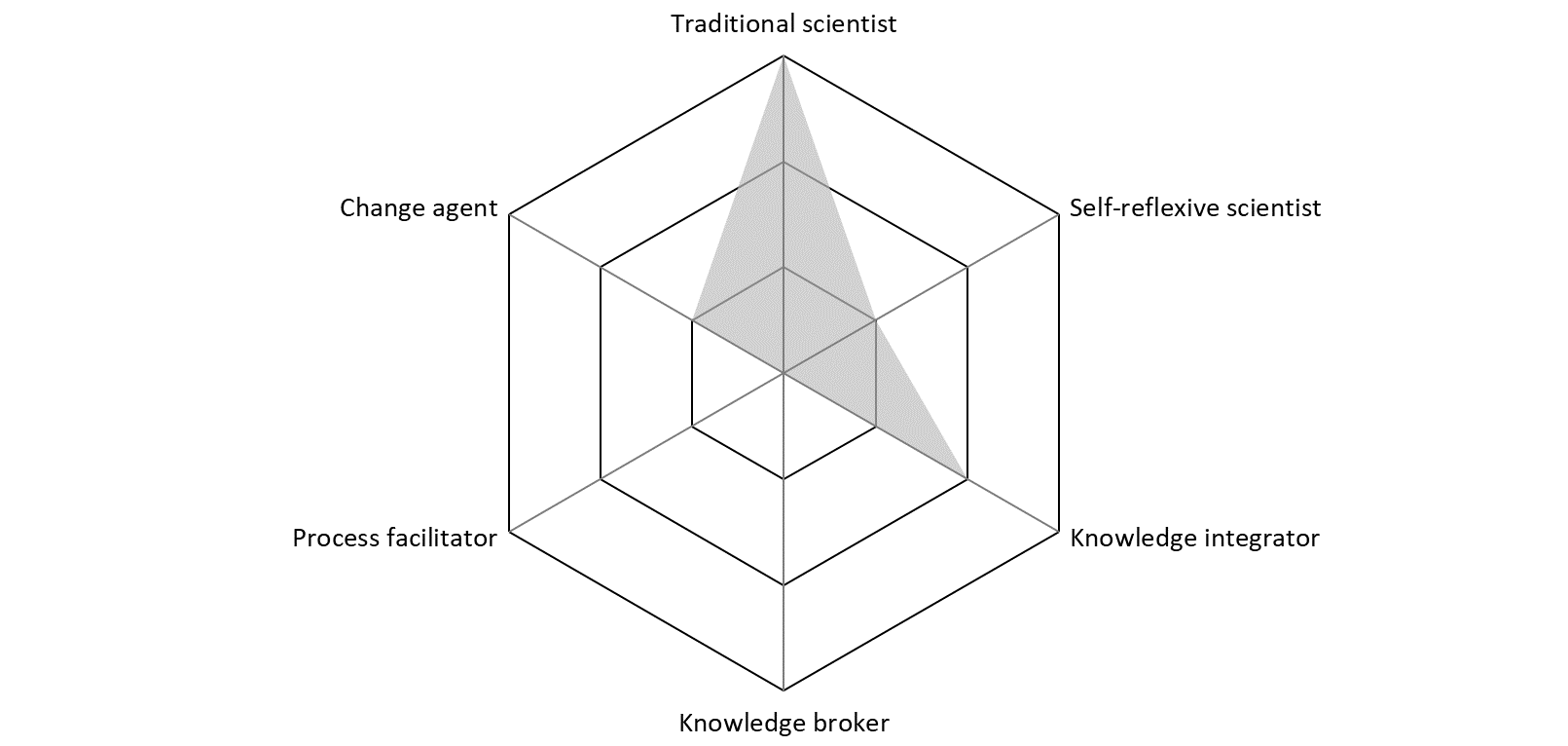


Researcher B.9
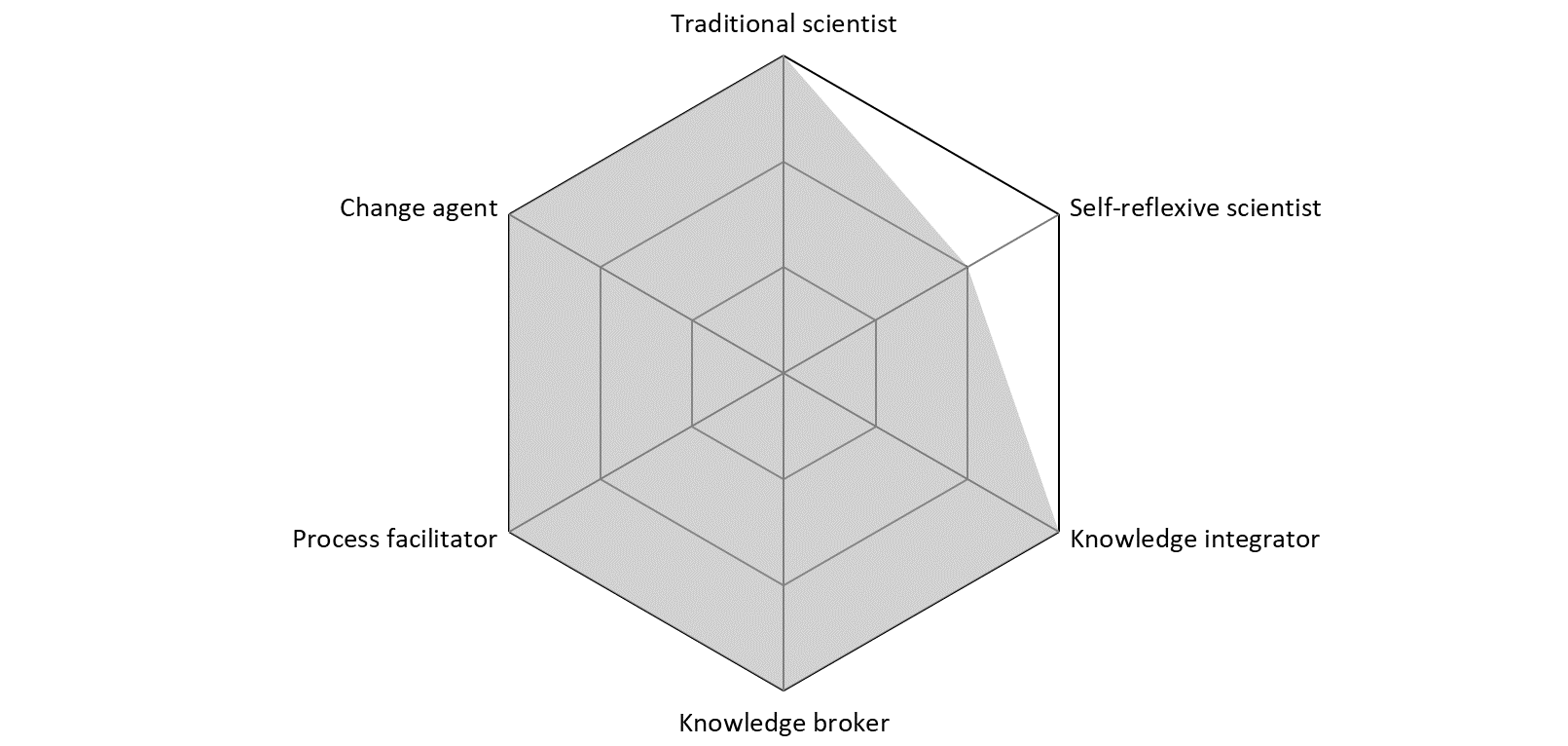


Researcher B.10
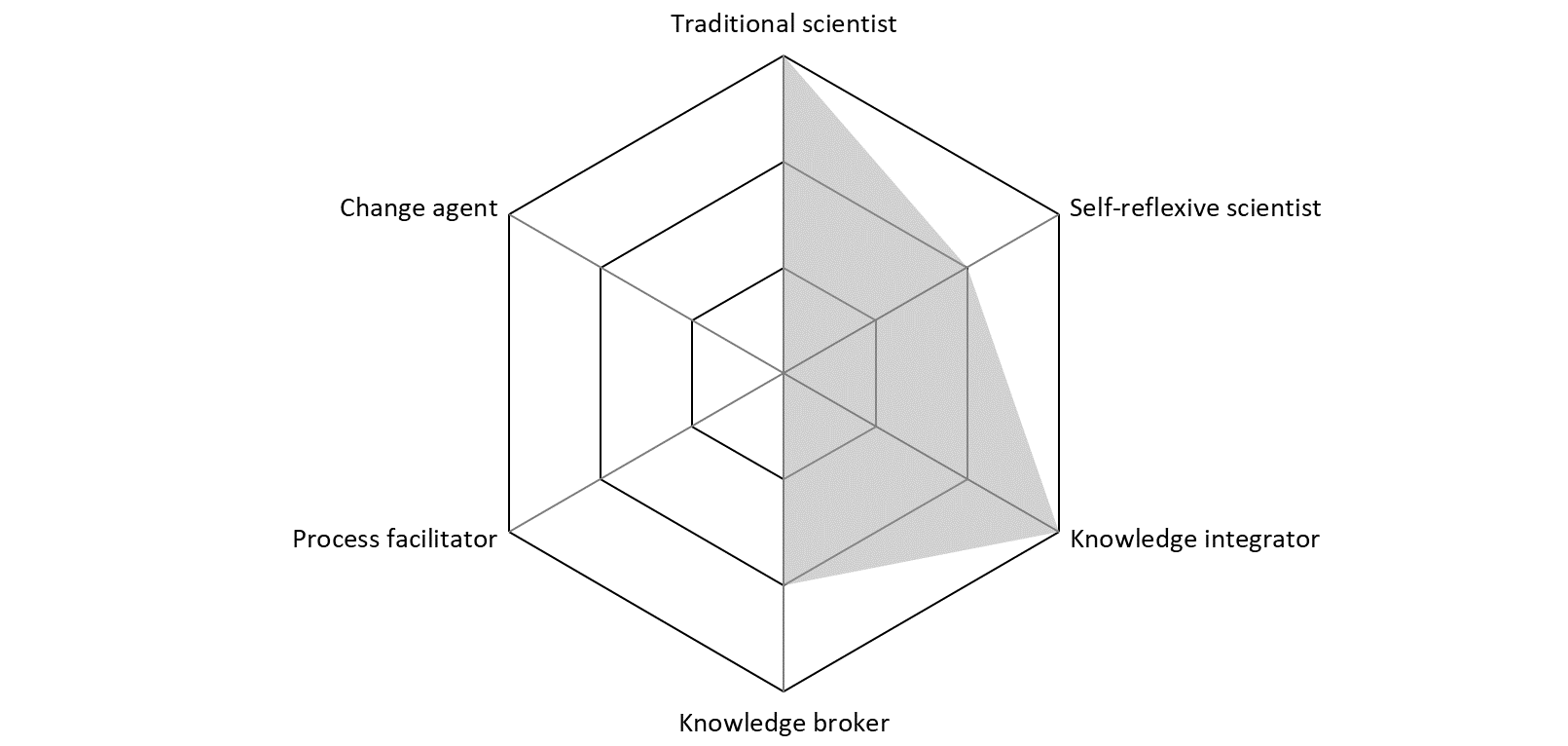


Researcher B.11
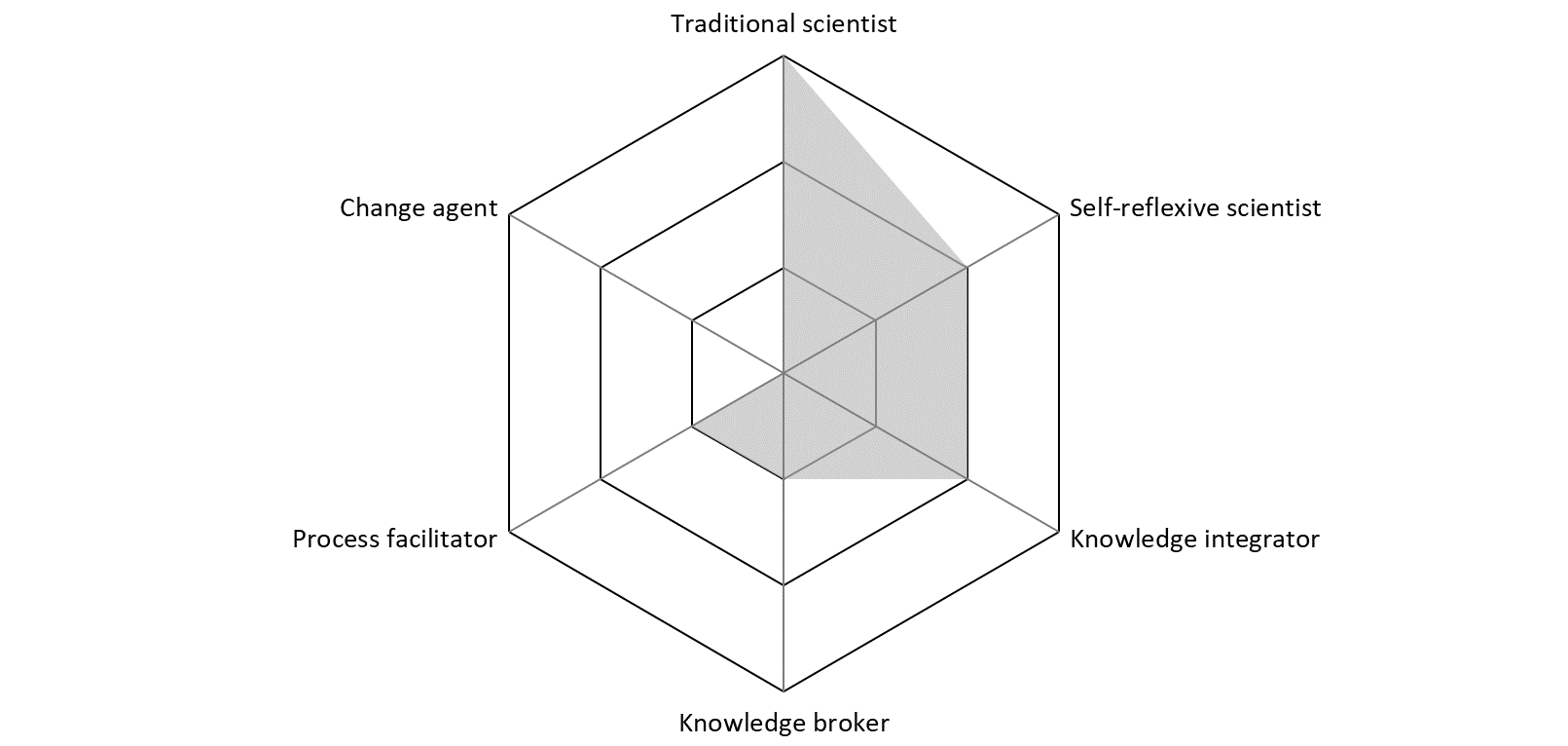


## Group 3

*(collected after workshop, not part of group discussion)*

Researcher B.12


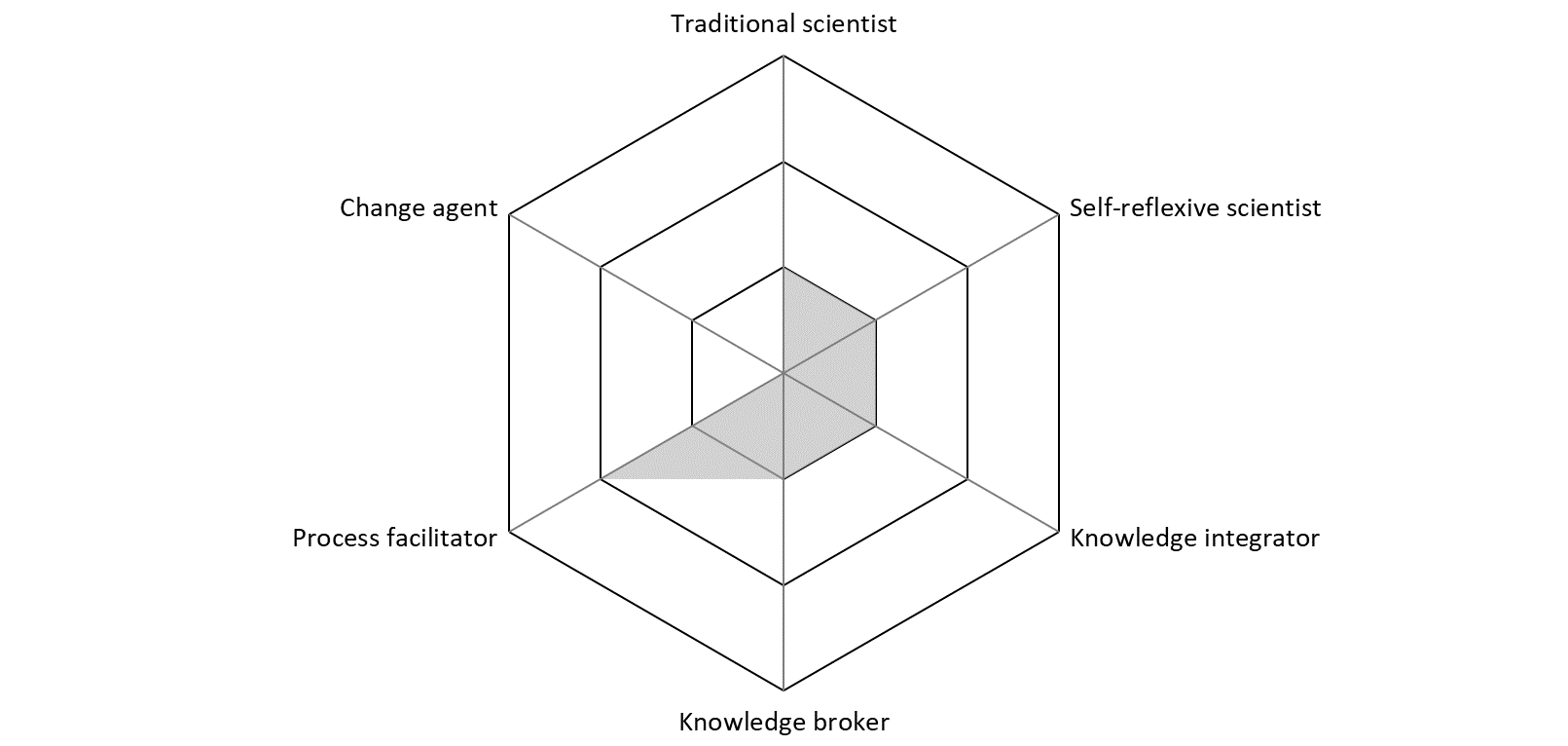

Researcher B.13


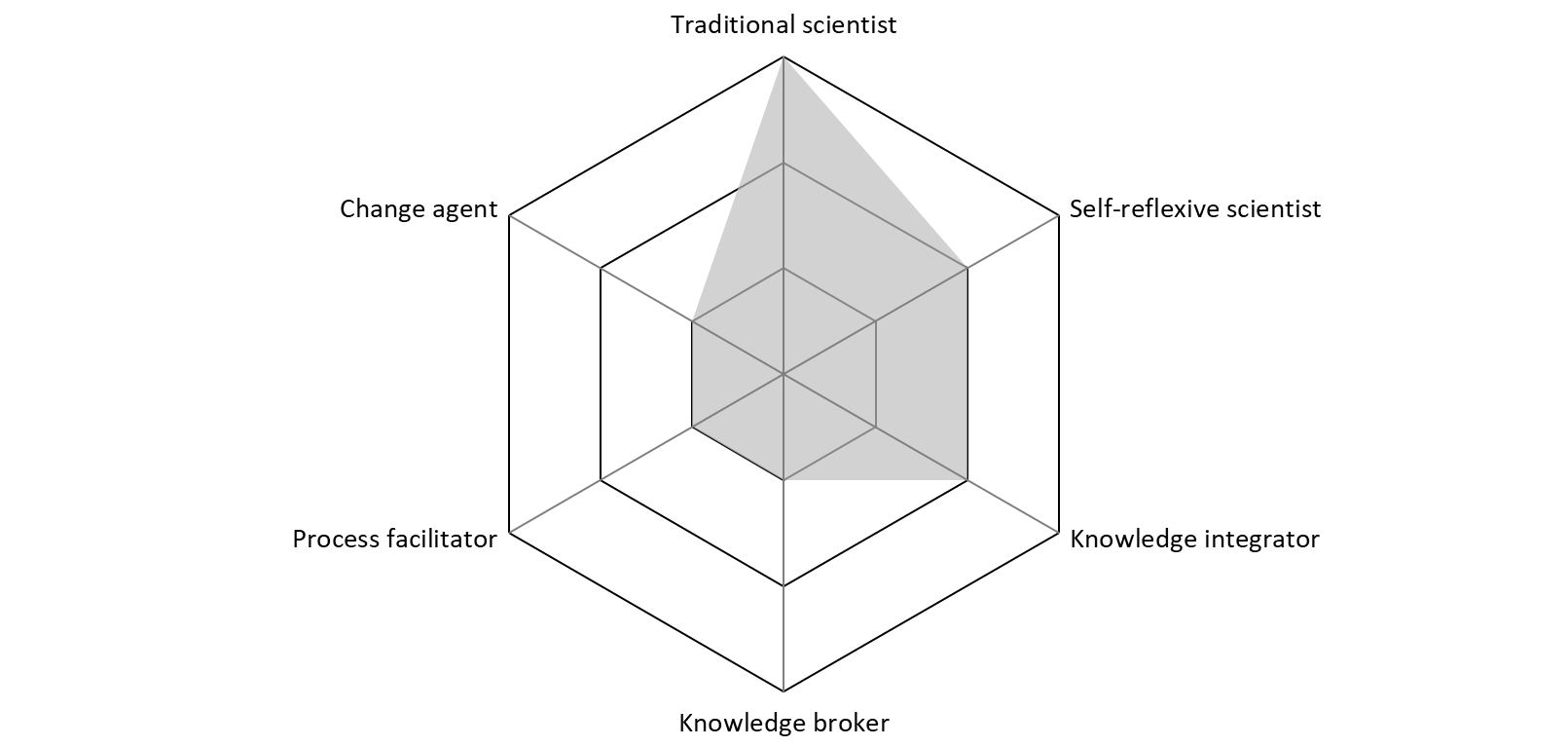


# Future improvement and application

During the discussions, participating researchers brought up some potentials for improvement of the tool, especially the role survey:

- The role of change agent was the most controversial regarding described activities and activities (not) included in the survey. Specifically, we recommend phrasing the first task as “proactively contribute to change in policy, practice, and/or the public sphere” instead of “intervene into policy and/or practice.”
- Despite focusing on a few key roles, the tasks of some roles may partly overlap in reality, especially between knowledge integrators and knowledge brokers (Hoffmann et al. 2022).
- While adding more roles would further decrease clarity, workshop participants mentioned that the role of project leader/manager was missing (Salomon 2023).
- Some participants considered the binary scoring of each activity too restrictive, but we see a trade-off here with the tool’s parsimony.
- Use of the term “researcher” instead of “scientist” throughout the survey and tool may be more inclusive. We also recommend using the label traditional researcher without any subtitle.
- Some participants also perceived a mismatch between the names of roles and the activities describing them, which may reflect differences between lay and expert understandings (Salomon 2023).

Note that such critique arising in the application of the tool is part of the desired reflection and does not decrease the tool’s usefulness if the facilitator manages to keep the discussions focused on the role profiles rather than the survey design.

From the application of the tool in two ITD projects, we identified several aspects for consideration in the future application of the tool in other project contexts:

- Researchers who want to apply the tool in their projects need to set aside enough time (ca. 1.5 hours) in (preferably in-person) project workshops.
- Responsibility for application of the tool should ideally be assigned in the project design phase or at the project start. Science integration experts are well-placed to use the tool and, for projects without such experts, project leaders may be an alternative.
- The tool can be adapted to the specific needs and interests of the ITD project. For instance, the roles included and the reflection questions can be amended, e.g., to make the tool applicable also to stakeholders in transdisciplinary research projects.
- It could be useful to provide input to participating researchers on how to deal with role challenges brought up in the reflection (cf. Bulten et al. 2021, 1280–81).
- We suggest thinking about potential follow-up activities that would address the opportunities and challenges identified in the reflection process, such as joint brainstorming of coping strategies.

# References

Bulten, Ellen, Laurens K. Hessels, Michaela Hordijk, and Andrew J. Segrave. 2021. “Conflicting Roles of Researchers in Sustainability Transitions: Balancing Action and Reflection.” *Sustainability Science* 16 (4): 1269–83. https://doi.org/10.1007/s11625-021-00938-7.

Hoffmann, Sabine, Lisa Deutsch, Julie Thompson Klein, and Michael O’Rourke. 2022. “Integrate the Integrators! A Call for Establishing Academic Careers for Integration Experts.” *Humanities and Social Sciences Communications* 9 (April):147. https://doi.org/10.1057/s41599-022-01138-z.

Salomon, Hanna. 2023. *Toward Resilient Ecosystems: An Analysis of the Opportunities and Challenges of Science Integration and Researcher’s Roles*. ETH Zürich: Master’s thesis.

1. Eawag: Swiss Federal Institute of Aquatic Science and Technology, Department of Environmental Social Sciences, Überlandstrasse 133, 8600 Dübendorf, Switzerland; [benjamin.hofmann@eawag.ch](mailto:benjamin.hofmann@eawag.ch) [↑](#footnote-ref-1)
2. ETH Zürich, Department of Environmental Systems Science, Zürich, Switzerland [↑](#footnote-ref-2)
3. ETH Zürich, Department of Environmental Systems Science, TdLab, Zürich, Switzerland [↑](#footnote-ref-3)
4. The survey is provided in the version we used for data collection and thus without the improvements suggested at the end of this document. [↑](#footnote-ref-4)
